# CDCA8 promotes bladder cancer survival by stabilizing HIF1α expression under hypoxia

**DOI:** 10.1038/s41419-023-06189-x

**Published:** 2023-10-09

**Authors:** Qiang Zhou, Wei Huang, Jing Xiong, Biao Guo, Xinghuan Wang, Ju Guo

**Affiliations:** 1 Department of Urology, First Affiliated Hospital of Nanchang University, Nanchang, China; 2 Department of Urology, Zhongnan Hospital of Wuhan University, Wuhan, China

**Keywords:** Bladder cancer, Cell growth

## Abstract

Hypoxia is an essential hallmark of solid tumors and HIF1α is a central regulator of tumor cell adaptation and survival in the hypoxic environment. In this study, we explored the biological functions of cell cycle division-related gene 8 (CDCA8) in bladder cancer (BCa) cells in the hypoxic settings. Specifically, we found that CDCA8 was significantly upregulated in BCa cell lines and clinical samples and its expression was positively correlated with advanced BCa stage, grade, and poor overall survival (OS). The expression of CDCA8 proteins was required for BCa cells to survive in the hypoxic condition. Mechanistically, CDCA8 stabilizes HIF1α by competing with PTEN for AKT binding, consequently leading to PTEN displacement and activation of the AKT/GSK3β signaling cascade that stimulates HIF1α protein stability. Significantly, HIF1α proteins bind to CDCA8 promoter for transcriptional activation, forming a positive-feedback loop to sustain BCa tumor cells under oxygen-deficient environment. Together, we defined CDCA8 as a key regulator for BCa cells to sense and prevail oxygen deprivation and as a novel BCa therapeutic target.

## Introduction

Bladder cancer (BCa) is one of the most commonly diagnosed urinary tumors with an annual 573,278 new cases and 212,536 deaths worldwide in 2020 [1]. Currently available BCa therapies include surgery, chemotherapy and immunotherapy that confer beneficial clinical outcomes. However, BCa inevitably relapses to become therapeutic resistant [2–4]. Recurrent BCa is in frequent association with the development of hypoxia, a typical feature of solid tumors. The HIF signaling is a key pathway for tumor cells to sense and prevail under low oxygen environment and HIF1α protein is a predominant effector in this process [5–7]. Mechanistically, hypoxia activates the AKT/GSK3β signaling cascade that enhances HIF1α protein stability and synthesis [8, 9]. Nuclear entry of accumulated HIF1α proteins enables its chromatin occupancy, driving the transcriptional network of hypoxia-regulated genes [10, 11]. Despite tremendous research efforts on BCa hypoxia in the past decades, additional key factor(s) are still continuously uncovered to provide new therapeutical routes.

The cell cycle division-related gene 8 (CDCA8) gene functions in cell division cycle and encodes a protein that is a component of the chromosome passenger complex (CPC). Temporal and spatial localization of the CPC in different organelles confers its precise regulatory functions in normal cell division during mitosis [12–16]. Recent evidences have established the connections of cell division cycle-related proteins and CPC complex with the tumorigenesis of a variety of human cancers [17–20]. In the current study, we identified CDCA8 overexpression in BCa cell lines and clinical cohorts, which bears diagnostic and prognostic values. By exploring the molecular mechanisms of CDCA8 actions, our study unlocked alternative BCa therapeutical targets.

## Results

### CDCA8 is overexpressed in BCa tissues and cells

**Fig. 1:**
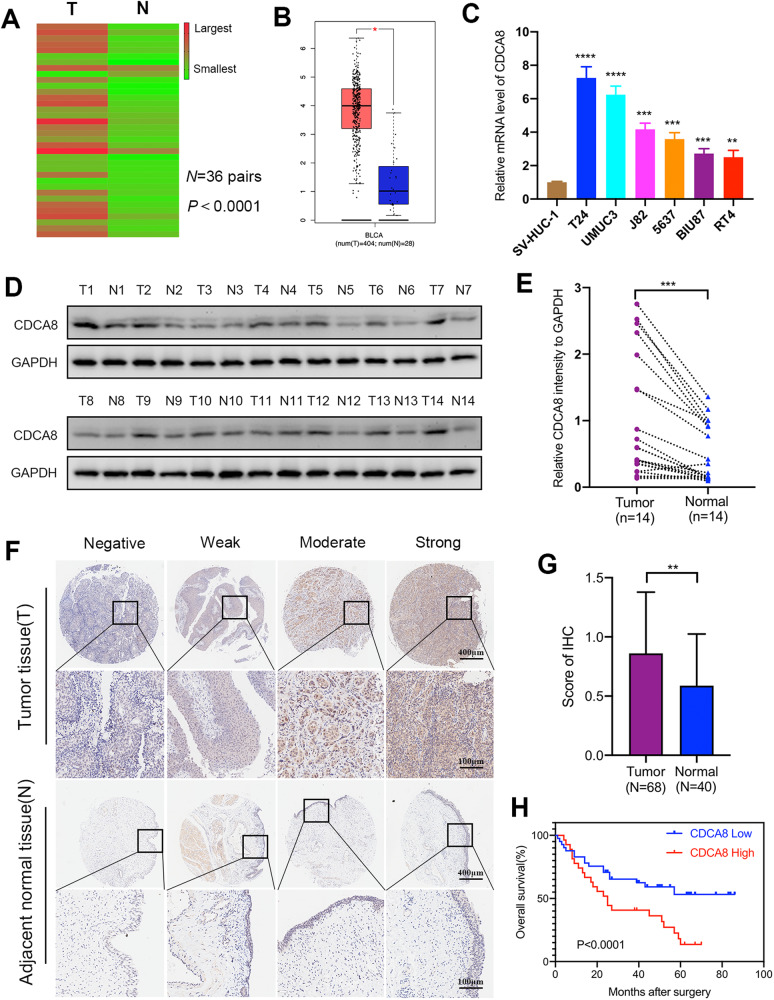
The expression and clinical significance of CDCA8 in BCa. **A** CDCA8 transcripts expression in 36 pairs of bladder tumor (T) tissues and adjacent normal bladder (N) tissues were displayed in a heat-map. **B** Overexpression of CDCA8 message was shown in BCa tissues based on analyses of the GEPIA database. **C** qRT-PCR assessment of CDCA8 mRNA expression in SV-HUC-1, T24, UMUC3, J82, 5637, BIU87 and RT4 cell lines. **D**, **E** Immunoblotting analysis of CDCA8 proteins in 14 pairs of T and N samples. **F** Tissue Chip assay to access CDCA8 protein expression in T and N samples. Representative pictures were elected to display negative, weak, moderate and strong CDCA8 IHC staining. **G** Average CDCA8 IHC scores to show its differential expression between T tissues and N tissues. **H** Kaplan-Meier profiling overall survival (OS) for CDCA8-high and CDCA8-low patients.

To profile BCa expression of CDCA8, we first carried out qRT-PCR test to assess its messages in 36 paired BCa tissues and adjacent normal bladder tissues and found that CDCA8 mRNA level was significantly higher in BCa tissues than normal controls (Fig. 1A). Similar results were obtained by analyses of CDCA8 transcripts in GEPIA database (Fig. 1B) and typical BCa cell lines (Fig. 1C). We next examined by Western blotting CDCA8 proteins in 14 paired BCa tissues versus adjacent normal bladder tissues. Consistently, CDCA8 proteins were significantly overexpressed in BCa tissues as compared to normal controls (Fig. 1D, E).

To further examine clinical CDCA8 expression in BCa cohorts, we performed IHC staining on a tissue Chip containing 68 BCa tissues and 40 adjacent normal bladder tissues. As shown, CDCA8 proteins were pronouncedly overexpressed in BCa tissues (Fig. 1F, G, Fig. S1). Specifically, in the 68 BCa cases, 41 cases had low CDCA8 expression and 27 cases had high expression (supplementary Table 1); and CDCA8 expression positively correlated with high BCa stage (*p* = 0.024) and advanced BCa grade (*p* = 0.031). Furthermore, the overall survival (OS) of 68 BCa patients was profiled with an average follow-up time of 35.6 months. Kaplan-Meier assay indicated OS of the CDCA8-low cohort was considerably superior to those CDCA8-high BCa patients (Fig. 1H). Importantly, by using univariate and multivariate Cox regression analyses, we identified CDCA8 as an independent prognostic factor for the OS of BCa patients (HR, 1.941; 95% CI, 1.003–3.757; *p* = 0.049) (Supplementary Table S2). Indeed, the mortality rate of CDCA8-high patients was 1.9 times of their CDCA8-low counterparts.

### CDCA8 promotes BCa cell survival in oxygen-deprived environment

**Fig. 2:**
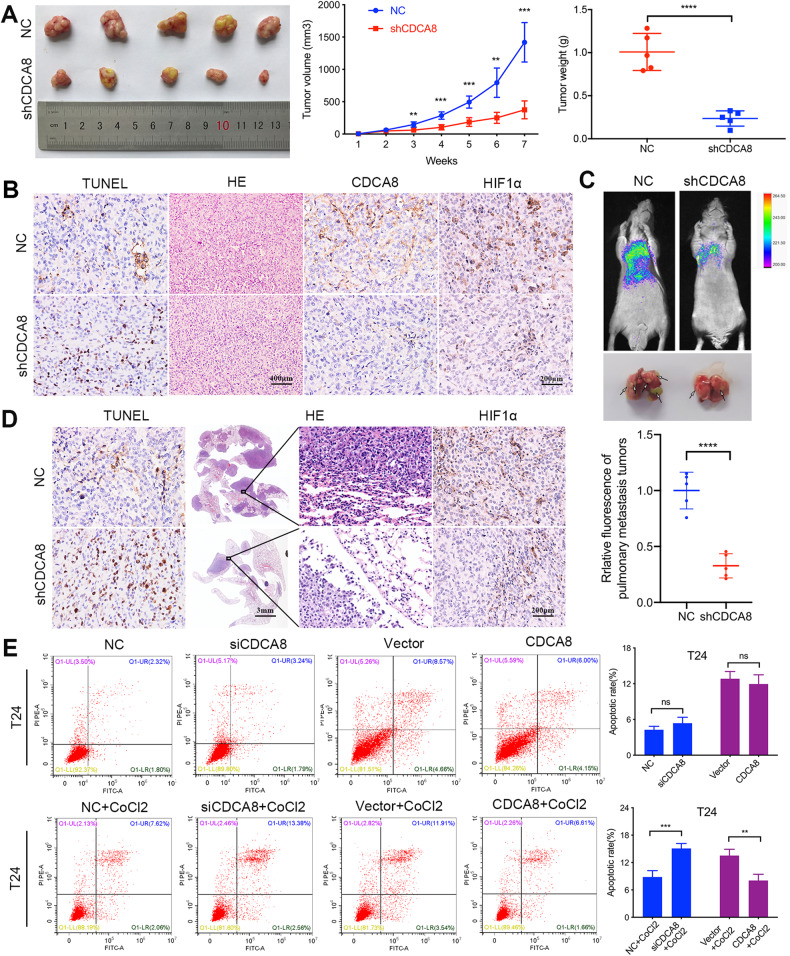
CDCA8 mediates BCa cells survival under hypoxia. **A** Tumor volume was observed every week, and the neoplasms growth curve was created. The weight of neoplasms was also weighed when the mice sacrificed. **B** The neoplasms were harvested for TUNEL staining to access cell apoptosis, H&E staining to detect nuclear atypia and IHC staining to evaluate CDCA8 and HIF1α expression. **C** Fluorescence assay of BCa pulmonary metastasis and statistical analysis of the fluorescence intensity. **D** The lungs of xenografted mice were isolated for TUNEL staining to assess cell apoptosis, H&E staining to measure nuclear atypia and IHC staining to monitor HIF1α expression. **E** Manipulation of CDCA8 expression to observe BCa cell apoptosis under normoxia and hypoxia, respectively.

In order to further explore the role of CDCA8 in affecting BCa biological behavior, we established a stable T24 BCa cell line in which CDCA8 was down-regulated (Supplementary Fig. S2). Then the stable BCa cells were transplanted into nude mice to generate the tumor model. Then the tumors were harvested after 7 weeks. As presented in Fig. 2A, compared with the negative control (NC) group, the growth and the average weight of neoplasm were obviously restrained in CDCA8 silenced group. More important, as shown in Fig. 2B, the mortality rate of tumor cells in CDCA8 knockdown group was considerably increased by using TUNEL staining, and the HE staining showed the degree of nucleus atypia in CDCA8 knock-down team was lower, in addition, we verified that CDCA8 was indeed hypo-expressed in the CDCD8 knockdown team. Similarly, we established the pulmonary metastasis model by injecting the stable BCa cells into the tail vein of mice, and the fluorescence intensity was detected after a 5-week feeding. As expected, the fluorescence intensity (Fig. 2C) of the CDCA8 knockdown group was markedly weaker than the NC group, similarly, the TUNEL staining data revealed higher mortality of BCa cells in interfering team, and the HE staining results also showed that the silenced group had fewer transferred cells (Fig. 2D).

Previous studies have documented the functional relevance of CDCA8 in tumor cell apoptosis [21, 22]. And first, to confirm the knockdown and overexpression efficiency, the qRT-PCR and Western blot analyses (Supplementary Fig. S3) were carried out after 48 hours transfection. However, to our surprise, under normoxia the change of CDCA8 expression had minimal or no effects on BCa cell viability (Fig. 2E). Next, we specifically addressed the effect of CDCA8 on BCa cells under the treatment of COCl2 to induce hypoxia. As shown in Fig. 2E, under the hypoxia CDCA8-KD significantly increased T24 cell apoptosis; in comparison, CDCA8 overexpression (OE) strongly improved T24 cell survival. We also observed similar results with the UMUC3 cells (a human transitional bladder carcinoma, Supplementary Fig. S3), affirming that CDCA8 plays a crucial role in tumor cells adaptation to the hypoxic environment. Significantly, by IHC staining of BCa xenograft tumors and pulmonary metastases, we found marked decline in HIF1α expression in the CDCA8-KD groups (Fig. 2B, D).

### Under hypoxic stress CDCA8 stabilizes HIF1α proteins via the AKT/GSK3β pathway

**Fig. 3:**
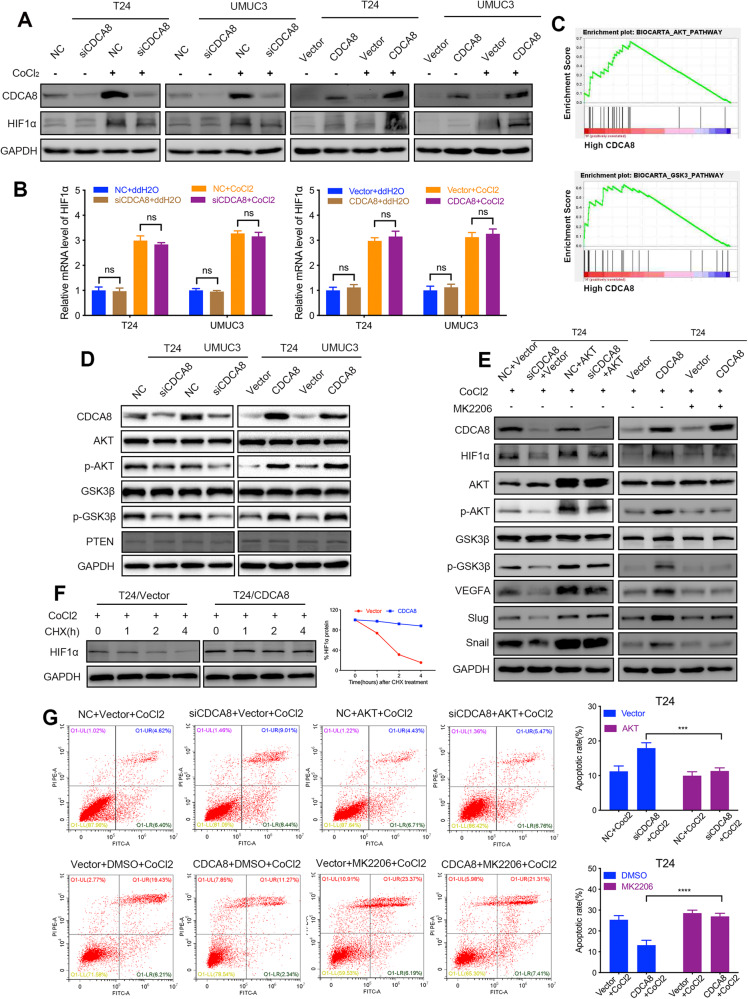
Under hypoxic stress CDCA8 stabilizes HIF1α proteins via the AKT/GSK3β pathway. **A**, **B** Manipulation CDCA8 expression (by silencing or overexpression) in BCa cells for immunoblotting and qRT-PCR analyses of effects on HIF1α protein expression under both normoxia and hypoxia, respectively. **C** GSEA test exposed enrichment of AKT/GSK3β pathway signatures in the CDCA8-high samples. **D** Immunoblotting assay to evaluate the impact of CDCA8-KD or OE on the AKT/GSK3β signaling pathway. **E** AKT-OE or inhibition in hypoxic T24 cells to evaluate the effects of CDCA8 manipulation (KD versus OE) on factors in the AKT/GSK3β networks, HIF1α and its targeted genes. **F** HIF1α degradation rate was determined by treating hypoxic T24 cells (control versus CDCA8-OE) with cycloheximide (CHX) (100 μg/ml). **G** Rescue experiments based on flow cytometry analysis of apoptosis in hypoxic T24 cells.

As shown above, we found that CDCA8 manipulation perturbed HIF1α protein expression in xenograft tumors. To directly address CDCA8 effects on HIF1α expression, we next knocked down CDCA8 in hypoxic BCa cells and found CDCA8-KD significantly decreased HIF1α protein expression while CDCA8-OE considerably increased HIF1α expression, respectively. Of significance, changes in HIF1α expression were less pronounced under normoxic conditions (Fig. 3A). Interestingly, either CDCA8-KD or OE did not alter the expression of HIF1α mRNA (Fig. 3B), suggesting CDCA8 regulates HIF1α expression at post-transcriptional level.

The AKT/GSK3β signaling cascade is known to increase the expression of HIF1α proteins [6, 23]; consistently, GSEA (gene set enrichment analysis) test indicated this pathway was indeed enriched in the CDCA8-high BCa samples (Fig. 3C). Based on these findings, we propose CDCA8 enhances HIF1α protein expression through activating the AKT/GSK3β pathway. In accordance, CDCA8-KD repressed AKT and GSK3β phosphorylation, while CDCA8-OE enhanced AKT and GSK3β phosphorylation (Fig. 3D). However, in these assays we observed no marked change in PTEN protein levels. Notably, we also found in both T24 and UMUC3 cells CDCA8-KD or OE motivated changes in HIF1α downstream factors under hypoxia, and these effects could be effectively reversed by AKT-OE or inhibition (with AKT inhibitor MK2206), respectively (Fig. 3E; Supplementary Fig. S4). Furthermore, protein half-life assay in T24 cells demonstrated that CDCA8-OE decreased HIF1α degradation rate (Fig. 3F). In addition, CDCA8-induced tumor survival was completely abrogated by AKT inhibitor MK2206 (Fig. 3G and Supplementary Fig. S5). Together, these results suggested CDCA8 mediates BCa cell adaptation to oxygen insufficiency by stabilizing HIF1α expression via activation of the AKT/GSK3β pathway.

### CDCA8 interacts with AKT proteins for activation

**Fig. 4:**
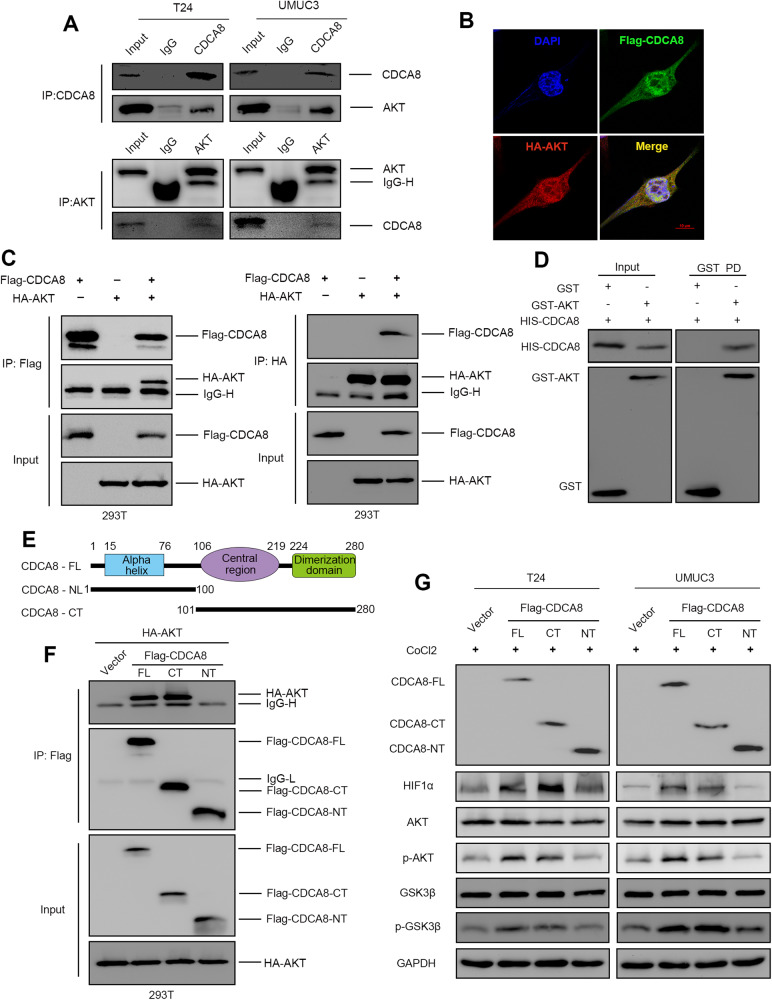
CDCA8 interacts with AKT for activation. **A** Co-IP assay displayed endogenous interaction between CDCA8 and AKT in T24 and UMUC3 cells. **B** Immunofluorescence confirmed CDCA8 and AKT were co-localized in UMUC3 cell. **C** Co-IP assay showed exogenous interaction between CDCA8 and AKT in 293 T cell. **D** GST-pulldown assay confirmed the direct interaction between CDCA8 and AKT in vitro. **E** General schematic diagram of CDCA8 domains. **F** Co-IP assay revealed interaction between CDCA8-CT and AKT in 293 T cells. **G** The overexpressed CDCA8-FL, CDCA8-NT and CDCA8-CT were transfected into T24 and UMUC3 cells, the protein level of HIF1α, the AKT phosphorylation and GSK3β phosphorylation in the group CDCA8-FL and CDCA8-NT were significantly upgraded.

We have evidenced that CDCA8 stabilizes HIF1a through activating the AKT/GSK3β pathway. To substantiate the mechanisms by which CDCA8 activates the AKT/GSK3β cascade to enhance HIF1a stability, we next performed an endogenous Co-immunoprecipitation (Co-IP) test and found that CDCA8 interacts with AKT in T24 and UMUC3 cells (Fig. 4A). Immunofluorescence (IF) staining also confirmed that CDCA8 and AKT co-localized in UMUC3 cells (Fig. 4B). Furthermore, exogenous Co-IP assay in 293 T cells also demonstrated the interactions between CDCA8 and AKT proteins (Fig. 4C). Additionally, GST-pulldown assay validated CDCA8-AKT direct interaction in the vitro settings (Fig. 4D). Next, we generated CDCA8 constructs (NT: N-terminus; CT: C-terminus) to specify the interaction domains by overexpression in 293 T cells (Fig. 4E). The followed Co-IP analysis revealed that both CDCA8 full-length (CDCA8-FL) and C-terminus (CDCA8-CT) interacted with AKT whereas its N-terminus (CDCA8-NT) had no binding with AKT (Fig. 4F). Significantly, overexpressing CDCA8-FL and CDCA8-CT (but not CDCA8-NT) in T24 and UMUC3 cells enhanced HIF1α protein expression and AKT and GSK3β phosphorylation (Fig. 4G). Collectively, these findings provided mechanistic insights that activation of the AKT/GSK3β cascade mediates HIF1α stabilization by CDCA8.

### CDCA8 and PTEN competes in AKT interactions

**Fig. 5:**
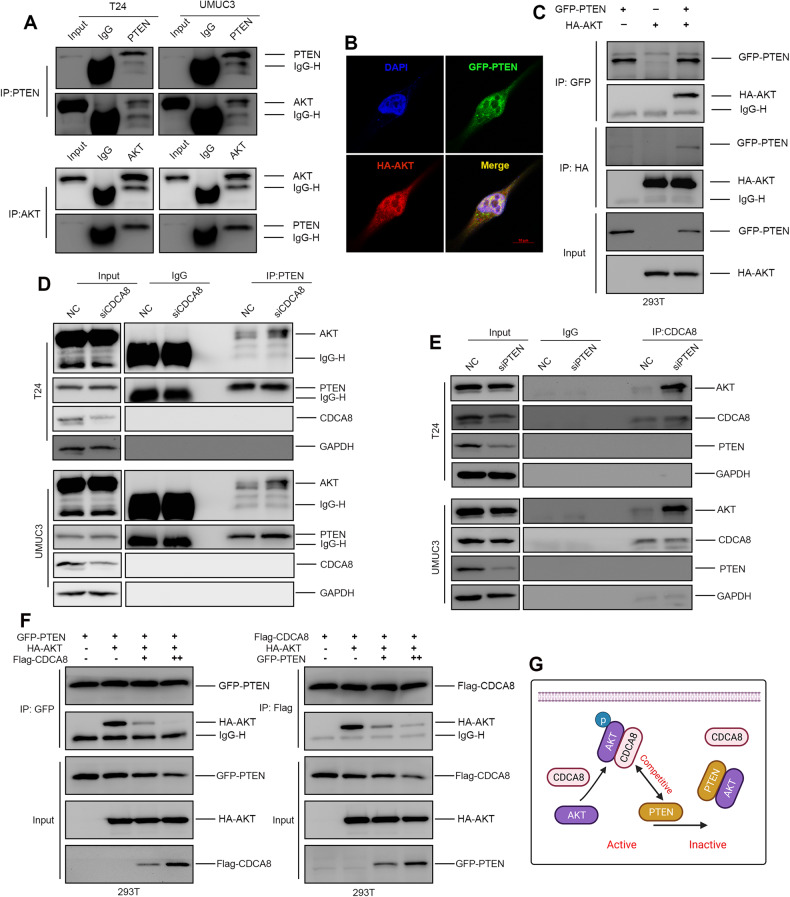
CDCA8 and PTEN competently interact with AKT. **A** Co-IP assay displayed endogenous PTEN-AKT interaction in T24 and UMUC3 cells. **B** IF assay confirmed PTEN and AKT co-localization in UMUC3 cell. **C** Co-IP assay showed exogenous PTEN-AKT interaction in 293 T cell. **D** CDCA8-KD enhanced PTEN-AKT interaction in T24 and UMUC3 cells. **E** PTEN interference increased CDCA8-AKT interaction in T24 and UMUC3 cells. **F** PTEN-AKT versus CDCA8-AKT interactions are mutually perturbed by CDCA8-OE versus PTEN-OE in 293 T cells, respectively. **G** A schematic diagram to display CDCA8-mediated AKT phosphorylation by displacing PTEN.

PTEN is a phosphatase that catalyzes AKT dephosphorylation and inactivation. In our endogenous Co-IP and IF staining assays, PTEN is found to interact with AKT for co-localization in both T24 and UMUC3 cells (Fig. 5A, B). PTEN-AKT interaction was also confirmed in exogenous expression and Co-IP assay in 293 T cells (Fig. 5C). However, we observed no direct interaction between CDCA8 and PTEN (Supplementary Fig. S7), suggesting that CDCA8 displaces PTEN for AKT binding and activation. Consistently, PTEN-AKT interaction was significantly enhanced by CDCA8-KD in both T24 and UMUC3 cells (Fig. 5D); and complementarily, in both T24 and UMUC3 cells PTEN-KD enhanced CDCA8-AKT interaction (Fig. 5E). In ectopic overexpression tests in 293 T cells, CDCA8-OE markedly reduced PTEN-AKT interaction while PTEN-OE substantially attenuated CDCA8-AKT interaction (Fig. 5F). Collectively, these results support the notion that CDCA8 and PTEN compete for AKT interaction and activation (Fig. 5G).

### HIF1α activates CDCA8 gene transcription by directly binding to its promoter

**Fig. 6:**
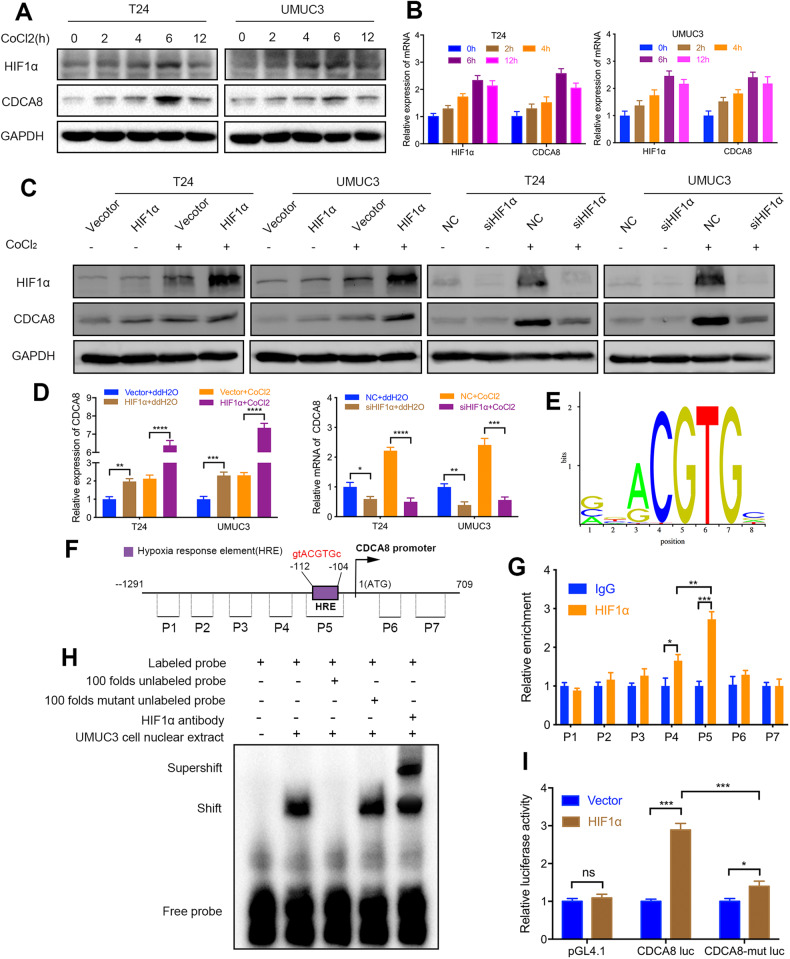
HIF1α binds to CDCA8 promoter and activates transcription. **A**, **B** Western blotting and qRT-PCR assays profiled changes in both HIF1α and CDCA8 proteins and transcripts along the time course of hypoxia, respectively. **C**, **D** Immunoblotting and qRT-PCR analysis accessed CDCA8 expression upon HIF1α manipulation (KD or OE) under normoxia and hypoxia, respectively. **E** A candidate HIF1α binding site was obtained by annotation of the JASPAR database. **F** Schematic drawing of the CDCA8 promoter with highlighted features. The promoter locus was marked as seven parts, together with indicated primers for ChIP-qPCR assay. **G** ChIP analysis of BCa UMUC3 cells upon transfection of vector (control) or Flag-tagged HIF1α. Processed chromatin was next immunoprecipitated with anti-Flag-HIF1α antibody for purification and quantification by qPCR analysis. **H** EMSA analysis. **I** Luciferase reporter assay in UMUC3 cells were conducted by co-transfection of HIF1α-expression vector (empty vector as control) with the following reporter constructs: pGL4.10-CDCA8-promoter, pGL4.10-CDCA8-promoter mutant, or pGL4.10 vector.

Next, we investigated the may underlying mechanism of CDCA8 overexpression in BCa cells. In the hypoxic environment, expression of HIF1α proteins was induced over the on-set of hypoxia time course, peaking at 4–6 h (Fig. 6A). Interestingly, CDCA8 expression correlated with HIF1α change profiling in both T24 and UMUC3 cells (Fig. 6A). Based on this finding, we speculate HIF1α transcriptionally regulates the CDCA8 gene in BCa cells. To test this hypothesis, we first monitored HIF1α and CDCA8 transcripts at a series of time points and found that their expression was indeed coupled (Fig. 6B). We then knocked down HIF1α and found in both normoxia and hypoxia HIF1α down-regulation led to marked repression of both CDCA8 mRNA and protein expression. In complementary assays, HIF1α-OE resulted in substantial increase in CDCA8 expression in both nomoxic and hypoxic settings (hypoxia conferred higher increase than the normoxia setting) (Fig. 6C, D).

Under hypoxia conditions HIF1α functions as a pivotal transcription factor to activate target gene promoters by binding to the core DNA sequence 5′-CGTG-3′ in the hypoxia response element (HRE). By computing human CDCA8 promoter in the GCBI database we indeed identified a potential HRE (5′-gtACGTGc-3′). This candidate HIF1α binding site was next validated by annotating the JASPAR database (Fig. 6E, F). Subsequently, to affirm HIF1α binding to CDCA8 promoter, we carried out ChIP-qPCR assay based on HIF1α precipitation. As shown in Fig. 6G, the P5 site on CDCA8 promoter had the highest Flag-HIF1α enrichment, followed by the P4 region. As controls, other test regions had no statistical significance in binding enrichment. We also designed another set of promoter fragment cloning of CDCA8, the ChIP assay also proved the binding site of HIF1α to CDCA8 promoter (Supplementary Fig. S8). Then, the EMSA was performed. A probe which contained a putative site was synthesized; DNA-protein complexes were detected when the probe was incubated with the nuclear extracts of UMUC3 cells. The results showed that an increased number of unlabeled probes decreased the band for the complexes, and HIF1α antibody also super-shifted the band for the complexes (Fig. 6H). Furthermore, luciferase reporter analysis was applied to determine whether HIF1α binding activates the CDCA8 promoter. As shown in Fig. 6I, compared with the control (empty vector), HIF1α-OE substantially enhanced the transcriptional activity of CDCA8 promoter. In contrast, a HRE mutant version (5′-caGTACAc-3′) was obviously abrogated of this transcriptional induction. Taken together, these findings demonstrated that CDCA8 and HIF1α reciprocally increase their expression, forming a positive feedback loop to sustain their levels under hypoxia.

## Discussion

Hypoxia is one of the important characteristics of solid tumors and one of the important mechanisms to promote the growth of solid tumors. When hypoxia occurs, tumor cells undergo a series of biobehavioral changes to adapt to the environment. Hypoxia is tightly associated to the tumor malignant progression, such as tumor cell proliferation, differentiation, apoptosis, phenotypic determination, angiogenesis, energy metabolism, resistance to treatment, and prognosis of patients [24].

CDCA8 protein functions in cellular mitosis and specifically, it is engaged in the stability of bipolar mitotic spindle [25]. The role of CDCA8 in cellular mitosis has been extensively documented; however, its functions in tumorigenesis is less explored. In this study, we disclosed CDCA8 overexpression in BCa, and its expression positively correlated with the stage and grade of tumor. Patients with high CDCA8 expression had a poor prognosis, exposing CDCA8 as an independent prognosis risk factor for BCa patients.

In addition, we defined CDCA8 as a crucial mediator of BCa cells’ responsiveness to oxygen shortage. Our results demonstrated that CDCA8 considerably drove the expression of factors in the HIF1α pathway, and under hypoxia, CDCA8 depletion would markedly down-regulated the expression of key components in the HIF1α pathway. In contrast, CDCA8 had negligible effect on the expression of HIF1α under normoxic conditions.

Mediated by AKT phosphorylation and activation, the AKT/GSK3β signaling pathway plays a crucial role in cellular processes like cell proliferation, transcription, migration, apoptosis, and glucose metabolism via [26–30]. It was extensively documented that AKT/GSK3β signaling pathway is abnormally overactivated by hypoxia in various tumors [8, 9]. However, the effectors of the activated AKT/GSK3β signaling pathway remain to be identified under the oxygen shortage conditions. Nevertheless, AKT signaling was found to mediate apoptosis resistance induced by hypoxia [31, 32]; and importantly, CDCA8 could activate the AKT/GSK3β pathway in tumors [33, 34]. Consistently, here we used GSEA test to validate the mechanistic link between CDCA8 expression and activation of the AKT/GSK3β pathway in BCa. Consistently, immunoblotting showed CDCA8 knock-down notably reduced both AKT and GSK3β phosphorylation; while CDCA8 overexpression had opposite effects. Significantly, in BCa the AKT/GSK3β signaling pathway functions in HIF1α expression. Indeed, AKT activation can reverse HIF1α pathway inhibition caused by CDCA8 knockdown; in comparison, AKT inhibition can recover the activation of HIF1a pathway mediated by CDCA8 upregulation. Additionally, in BCa changes of CDCA8 expression impacted cell apoptosis under hypoxia, while these effects can be overcome by AKT pathway activation or inhibition. Together, these findings supported the AKT/GSK3β pathway mediates CDCA8 effects on HIF1α protein stabilization rather than HIF1a gene transcription.

To further explored the mechanistic links between CDCA8 expression and AKT phosphorylation, we launched a series of tests to expose direct CDCA8-AKT interaction. PTEN functions to impede AKT phosphorylation by converting PIP3 into PIP2 and here we showed the direct PTEN-AKT interaction in BCa cells. Significantly, we observed no direct interaction between PTEN and CDCA8. However, CDCA8-AKT complex was attenuated by PTEN, and conversely, CDCA8 also competes with PTEN for AKT binding. Together, these findings supported the notion that in BCa AKT phosphorylation correlates with the equilibrium with CDCA8 and PTEN

HIF1α is a predominant transcription factor in development of hypoxia adaptation and tolerance in tumor cells. In accordance, in hypoxic environment the HIF1α transcriptional networks underlay tumor occurrence and development [35]. Interestingly, by JASPAR prediction we found that CDCA8 is also a HIF1α target gene. Indeed, we determined HIF1α directly binds to CDCA8 promoter for transactivation in BCa cells. Thus, CDCA8 and HIF1α form a positive-feedback loop to co-sustain the AKT signaling.

**Fig. 7:**
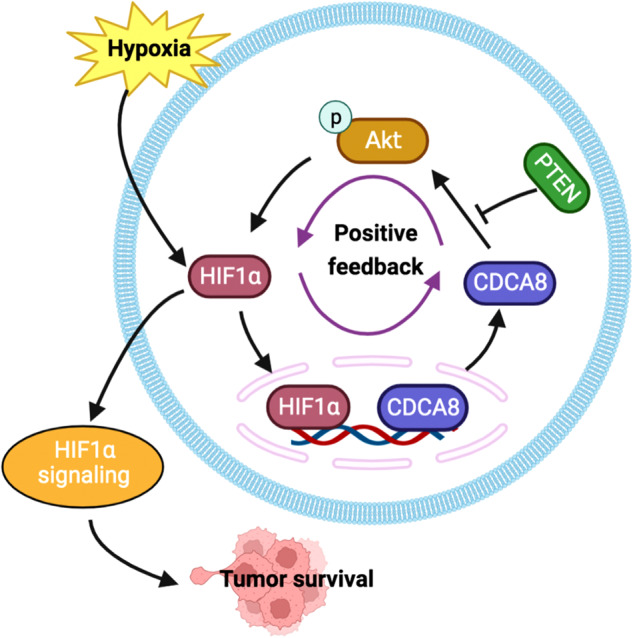
A proposed model on CDCA8 functions in BCa cell survival under an anoxic condition. The anoxic microenvironment features solid tumors including BCa. We reported that under hypoxia CDCA8 displaces PTEN from AKT, leading to activation of the AKT/GSK3β cascade for HIF1α protein stabilization. In turn, HIF1α proteins bind to CDCA8 gene promoter to drive its transcription. Collectively, we defined CDCA8 and HIF1α form a positive-feedback loop that sandwiches with the AKT/GSK3β pathway.

In summary, as shown in Fig. 5H and Fig. 7, we report CDCA8 competes with PTEN for AKT binding, leading to stabilization of HIF1α proteins, which in turn transcriptionally activate CDCA8 gene expression. The identification of a novel positive-feedback loop singled out CDCA8, in combination with other drug targets, such as AKT inhibitors and HIF1α inhibitors, as a novel BCa therapeutic targets.

## Materials and methods

### Cell line and cell culture

Cell lines in this study include six human BCa lines (T24, UMUC3, J82, 5637, BIU87, and RT4), a human bladder epithelial cell line (SV-HUC-1), and a human embryonic kidney cell line (293 T). All cell lines were provided by the Stem Cell Bank, Chinese Academy of Sciences in Shanghai and have recently been authenticated. RPMI-1640 medium was used for SV-HUV-1, T24, 5637, BIU87 and RT4 cell culture, DMEM medium was applied for UMUC3 and 293 T cell culture, and MEM medium was utilized for J82 cell culture. All culture mediums were freshly prepared with 10% fetal bovine serum (FBS). To simulate the hypoxic conditions, cells were subjected to a 4-h pretreatment of CoCl2 (100 μM, Sigma c8661) prior to collection [36, 37].

### Tissue samples

BCa tissues and adjacent normal bladder tissues (*n* = 36) applied in this research were obtained from BCa patients in the First Affiliated Hospital of Nanchang University. And the current research was approved by the Ethics Committee of the First Affiliated Hospital of Nanchang University with all individuals informed consent. The tissue chip (XT18-040, 68 BCa tissues and 40 adjacent normal bladder tissues) was purchased from Shanghai Outdo Biotech Company.

### RNA extraction and qRT-PCR

These assays were performed as previously described [38]. The primers sequence for CDCA8, HIF1α, and GAPDH were listed as below: CDCA8-F: 5′-GCAGGAGAGCGGATTTACAAC-3′, CDCA8-R: 5′-CTGGGCAATACTGTGCCTCTG-3′; HIF1α-F: 5′-GAACGTCGAAAAGAAAAGTCTCG-3′, HIF1α-R: 5′-CCTTATCAAGATGCGAACTCACA-3′; GAPDH-F: 5′-GGAGCGAGATCCCTCCAAAAT-3′, GAPDH-R: 5′-GGCTGTTGTCATACTTCTCATGG-3′.

### siRNAs, plasmids and shRNA

The siRNAs (siCDCA8: 5′-GGUUUGACUCAAGGGUCUUTT-3′, siPTEN: 5′-ATCGATAGCATTTGCAGTATA-3′, and si HIF1α: 5′-GCCGCUCAAUUUAUGAAUATT-3′) to interfere CDCA8, PTEN and HIF1α respectively were obtained from Shanghai GenePharma Co., Ltd. A 3× Flag-pcDNA3.1 vector was cloned into the cDNAs of CDCA8-FL, CDCA8- NT, and CDCA8-CT. AKT cDNA were cloned into a HA-pcDNA3.0 vector. A GFP-pcDNA3.1 vector was cloned into the cDNA of PTEN. The shCDCA8: 5′-GGTTTGACTCAAGGGTCTT T-3′, and shNC: 5′-TTCTCCGAACGTGTCACGT-3′ were also obtained from GenePharma Co., Ltd. The sequence of plasmids primer is available on request.

### HE, IHC, immunofluorescence and TUNEL staining

These assays were performed as previously described [38]. And in the case of CDCA8 staining, intensity was scored as 0, 1, 2, or 3, corresponding to achromaticity, light yellow, pale brown, and sepia. In addition, the percentage score was defined as follows: 0 to 5%, 0 points; 6 to 25%, 1 point; 26 to 50%, 2 points; 50 to 75%, 3 points; and 76 to 100%, 4 points. A final histochemical score was calculated by multiplying the intensity score by the percentage score. The ultimate staining scores were categorized as 0 (0), 0.5 (1, 2), 1(3, 4), 1.5 (6, 9), and 2 (12). These scored as 0, 0.5, 1 were defined as the low expression group and scored as 1.5, 2 were defined as the high expression cohort.

### Flow cytometry analysis

The cell apoptosis analysis was carried out according to the manufacturer’s instruction of FITC/PI apoptosis detection kit (Cat. #558547, BD) with flow cytometry.

### Immunoblot analysis and reagents

The collected samples were lysed on ice with RIPA buffer which added phosphatase inhibitor and protease inhibitor. The protein lysates were isolated by SDS-PAGE and immunoblot analysis was performed. The antibodies were listed. Antibodies against AKT (4685, CST), p-AKT (4060 L, CST), CDCA8 (sc-376635, Santa Cruz), GSK3β (12456 S, CST), p-GSK3β (9323, CST), HIF1α (NB100-105, Novus Biologicals), PTEN (9559 S, CST), Snail (3879, CST), Slug (7585, CST), VEGFA (50661, CST), GAPDH (sc-365062, Santa Cruz), Flag tag (A4596, Sigma), HA tag (TA180128-1, OriGene), GFP tag (ab290, Abcam), Mouse-IgG (10283-1-AP, Proteintech), Rabbit-IgG (10284-1-AP, Proteintech), MK2206 (S1078, Selleck), were purchased from indicated companies.

### Co-immunoprecipitation (Co-IP) analysis

Magnetic beads in conjugation with the corresponding antibody were prepared by incubation at 4 °C for 4 h with slow shaking. After 3x washing with binding buffer, beads were subjected to immunoprecipitation reaction by incubation with cell lysate for overnight rotation at 4 °C. Next day, the immunocomplexes were washed 3 times before elution with 1× SDS buffer. Subsequently, the collected samples were submitted to immunoblotting analysis.

### GST pull-down analysis

GST-fused protein was pre-incubated with glutathione–Sepharose beads, followed by incubation with recombinant His-tagged proteins at 4 °C for 2 h. Then the beads were eluted with GST-binding buffer and the elutes were resolved by SDS-PAGE for immunoblotting assay.

### Chromatin immunoprecipitation (ChIP) and luciferase analysis

ChIP analyses were carried out based on manufacturer’s instructions of the SimpleChIP® Kit (Cat. #56383, CST). Briefly, T24 cells were transfected with 3× Flag-pcDNA3.1-HIF1α or empty vector (negative control). Cells were next subjected to cross-linking, chromatin shearing, and nuclear extraction. Then immunoprecipitation was conducted with anti-Flag antibody and precipitated chromatin was subjected to de-crosslinking, purification, and quantification analyses by qRT-PCR.

For luciferase reporter analysis, T24 cells were co-transfected with empty vector (negative control) or HIF1α expression vector, together with the following reporter constructs: pGL4.1-CDCA8-promoter, pGL4.1-CDCA8-promoter mutant (MUT), or pGL4.1-empty vector. After 48-h of cell culture, luciferase activity was measured using Luc-Pair TM Duo-Luciferase Assay Kit 2.0 (GeneCopoeial lnc, USA).

### Electrophoretic mobility shift assay (EMSA)

Briefly, nuclear extracts were obtained, synthetic biotinlabeled oligonucleotides were synthesized. The probe of was subsequently incubated with nuclear extract or purified protein. The DNA-protein complex was separated, and detected with HRP-conjugated streptavidin.

### Xenograft model and pulmonary metastasis model

Manipulations of the BCa xenograft model and pulmonary metastasis model were conducted as previously described [38]. Briefly, 1 × 10^6^ T24-shCDCA8 and T24-shNC (control) cells were injected into the subcutaneous flank or the tail vein of 4-week-old male BALB/c-nude mice, respectively. Tumor size and weight were monitored and fluorescence intensity was detected after 5-week of feeding. Collected samples were further analyzed with H&E, IHC and TUNEL staining assays. All animal experiments were performed with the approval of the Animal Experimentation Ethics Committee of the First Affiliated Hospital of Nanchang University.

### Statistical analysis

All statistical analyses were conducted with the IBM SPSS software program (version 23.0). Each experiment was based on three repeats. Student’s t test and chi-square test were used to calculate statistical significance. Kaplan-Meier test was used for overall survival (OS) assessment using the log rank test. The hazard ratio (HR) and 95% confidence interval (CI) were calculated with the Cox proportional hazards regression model. P-values of < 0.05 in two-sided assays was set as statistical significance.

### Supplementary information

Supplemental Material

Original Data File

## Data Availability

The data are available from the corresponding author on reasonable request.
