# Supplementary material for: CDCA8 promotes bladder cancer survival by stabilizing HIF1α expression under hypoxia
Source: Cell Death Dis. 2023 Oct 9;14(10):658. doi: 10.1038/s41419-023-06189-x (PMC10562466; doi:10.1038/s41419-023-06189-x)
Supplement: Supplementary file 1 — Supplemental Material [file 41419_2023_6189_MOESM1_ESM.docx]

**CDCA8 promotes bladder cancer survival by stabilizing HIF1α expression** **under** **hypoxia**

| Variable | Total(N=68) | CDCA8 low (n=41) | CDCA8 high (n=27) | *p* value |
| --- | --- | --- | --- | --- |
| Gender, n (%) |  |  |  | 0.471 |
| Male | 58 (85.3) | 36 (87.8) | 22 (81.5) |  |
| Female | 10 (14.7) | 5 (12.2) | 5 (18.5) |  |
| Age (year), n (%) |  |  |  | 0.055 |
| ＜65 | 23 (33.8) | 13 (31.7) | 10 (37.0) |  |
| ≥65 | 45 (66.2) | 28 (68.3) | 17 (63.0) |  |
| Tumor stage,n (%) | |  |  | 0.024 |
| Ta, T1, T2 | 45 (66.2) | 32 (78.0) | 14 (51.9) |  |
| T3, T4 | 23 (33.8) | 9 (22.0) | 13 (48.1) |  |
| Tumor grade, n (%) | |  |  |  |
| G1、G2 | 23 (33.8) | 18 (43.9) | 5 (18.5) | 0.031 |
| G3 | 45 (66.2) | 23 (56.1) | 22 (81.5) |  |
| Tumor size(cm), n (%) | |  |  | 0.732 |
| ≤5 | 54 (79.4) | 32 (78.0) | 22 (81.5) |  |
| ＞5 | 14 (20.6) | 9 (22.0) | 5 (18.5) |  |
| Multiplicity of  tumor, n (%) | |  |  | 0.675 |
| No | 59 (86.8) | 35 (85.4) | 24 (88.9) |  |
| Yes | 9 (13.2) | 6 (14.6) | 3 (11.1) |  |

**Supplementary table 1.** Correlation between CDCA8 expression and clinical features of patients.

*The value = mean ± standard deviation.

**Supplementary table 2.** Univariable and multivariable analysis for overall survival of patients with bladder cancer.

| Variable | Univariate | | Multivariate | |
| --- | --- | --- | --- | --- |
|  | HR (95%CI) | *P*值 | HR (95%CI) | *P*值 |
| Age (≥65 vs＜65) | 1.088 (0.558-2.119) | 0.805 | - | - |
| Gender (Male vs Female) | 0.500 (0.227-1.102) | 0.085 | - | - |
| Tumor size  (＞5 vs ≤5) | 0.697 (0.307-1.583) | 0.389 | - | - |
| Multiplicity of  tumor (Yes vs No) | 1.050 (0.410-2.691) | 0.919 | - | - |
| Tumor stage  (T3, T4 vs Ta, T1, T2) | 2.208 (1.168-4.171) | 0.015 | 1.834 (0.946-3.555) | 0.073 |
| Tumor grade  G3 vs G1, G2 | 1.128 (0.571-2.228) | 0.729 | - | - |
| CDCA8 status  (High vs Low) | 2.267 (1.200-4.267) | 0.012 | 1.941 (1.003-3.757) | 0.049 |

HR=hazard ratio; CI=confidence interval.


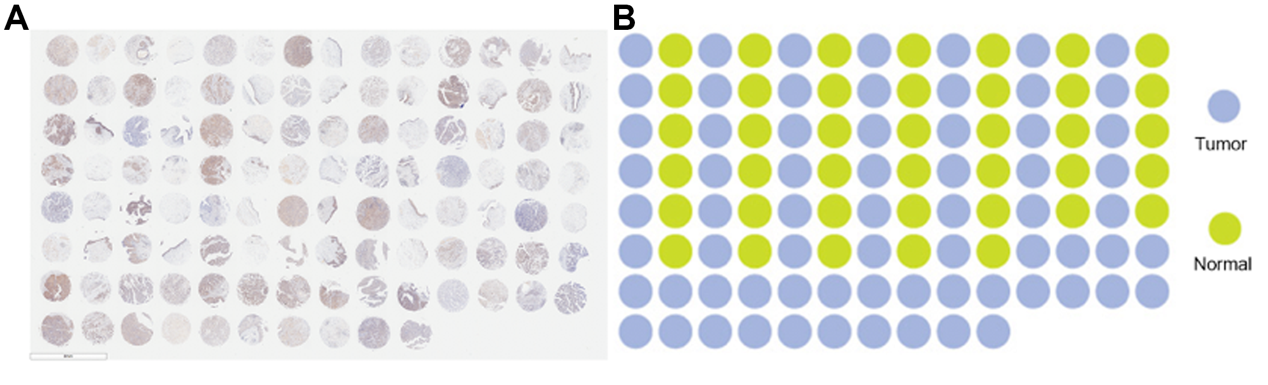


**Supplementary Fig. S1** (1) The IHC of the tissue chip including 68 BCa tissues and 40 adjacent normal bladder tissues. (2) General schematic diagram of the tissue chip.

**
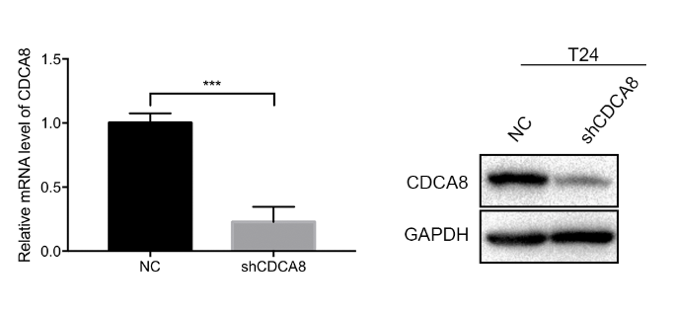
**

**Supplementary Fig. S2** qRT-PCR and Western blot analyses verified the knock down efficiency of CDCA8 in stable cells

**
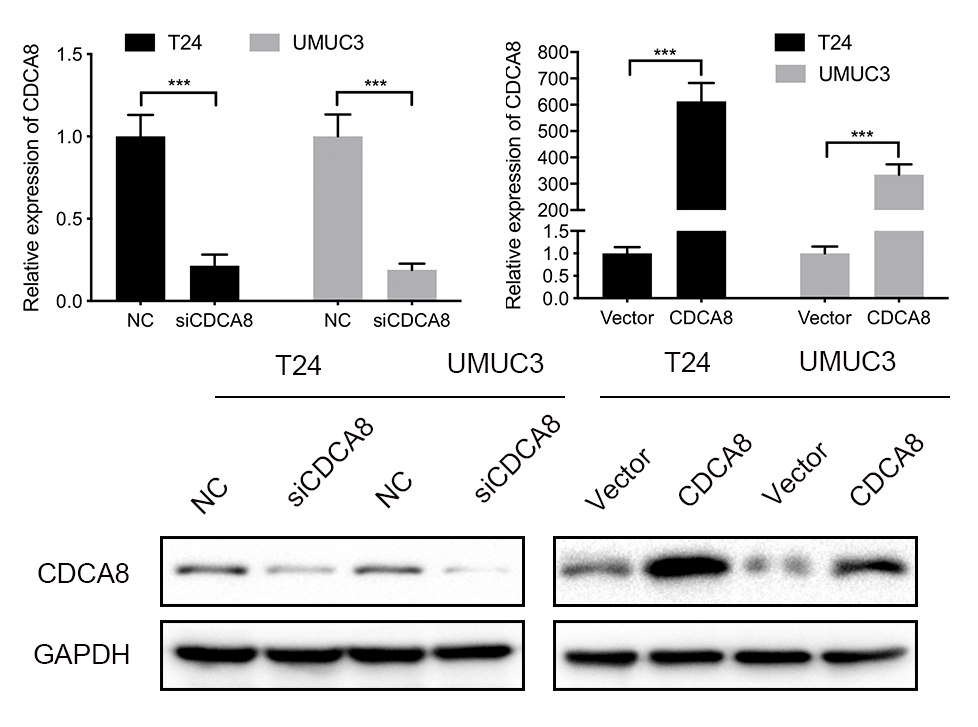
**

**Supplementary Fig. S3** qRT-PCR and Western blot analyses affirmed the interfering and upregulated efficiency of CDCA8 in relevant BCa cells (T24 and UMUC3).

**
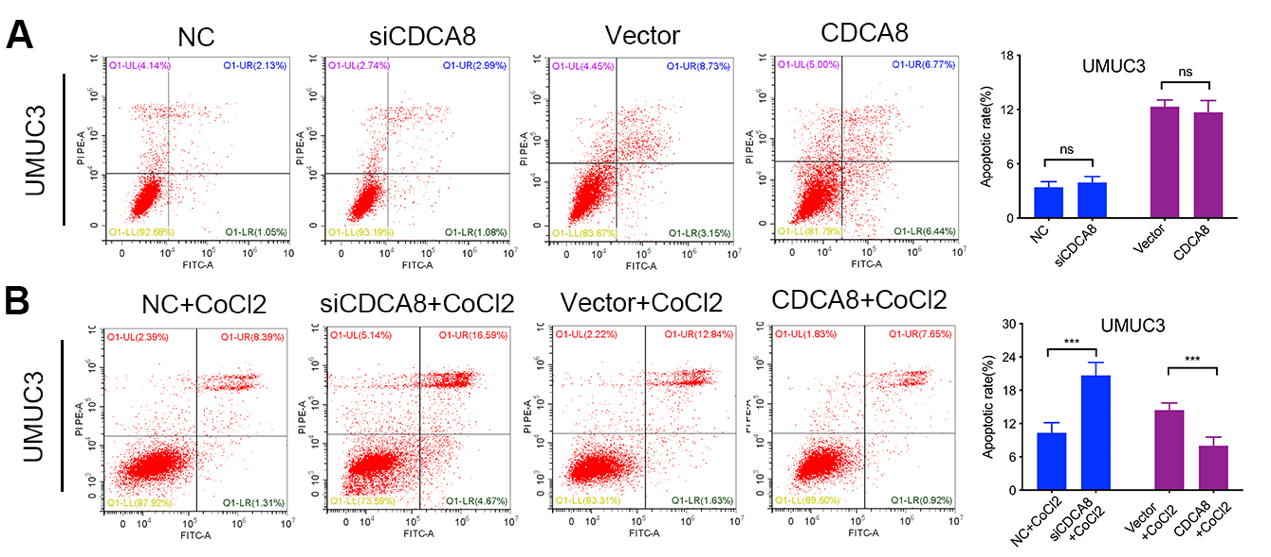
**

**Supplementary Fig. S4** (A)The expression of CDCA8 affected the apoptosis of UMUC3 cells under normoxia. (B)The expression of CDCA8 affected the apoptosis of UMUC3 cells under hypoxia.

**
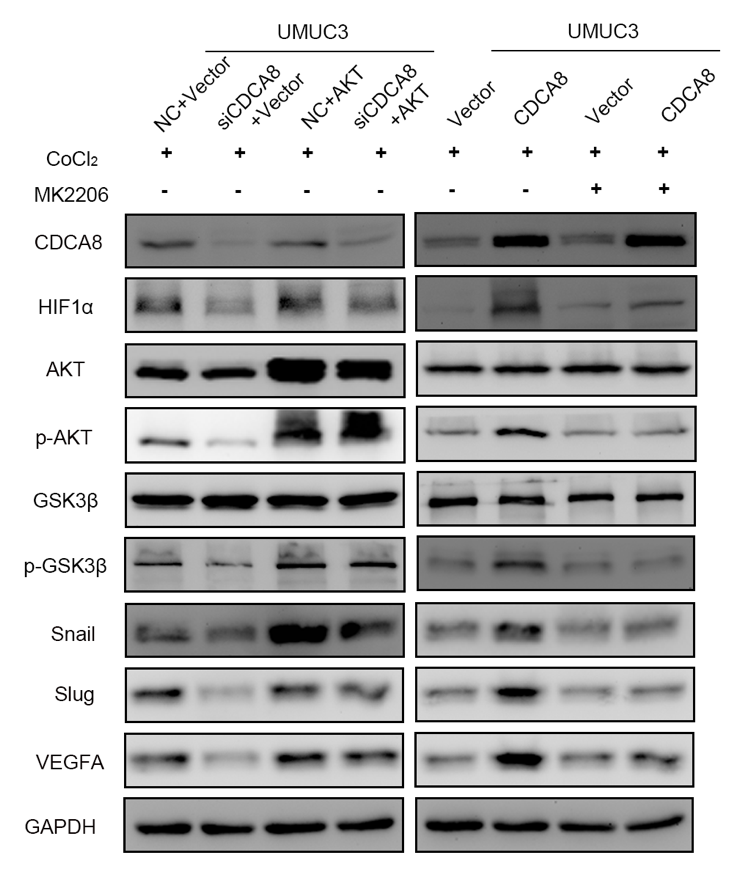
**

**Supplementary Fig. S5** AKT overexpression and inhibitor evaluate AKT/GSK3β signaling pathway related factors, HIF1α and its targeted genes in UMUC3 cells caused by CDCA8 deficiency and elevating under hypoxia.

**
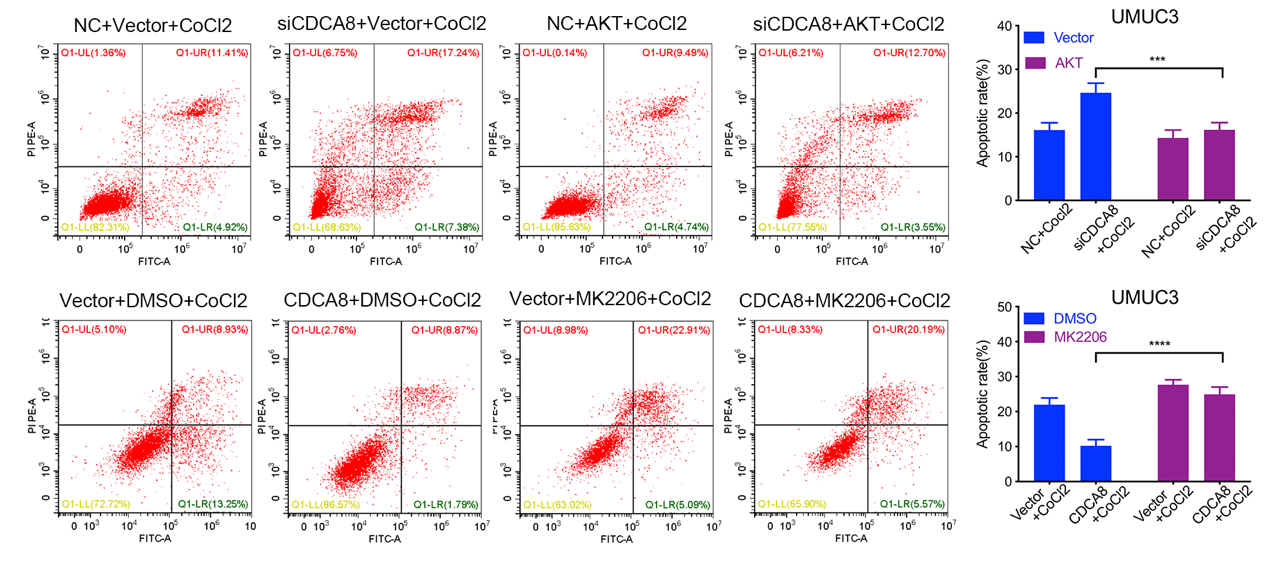
**

**Supplementary Fig. S6** Rescue experiments of flow cytometry analysis to detect cell viability in UMUC3 cells under hypoxia.

**
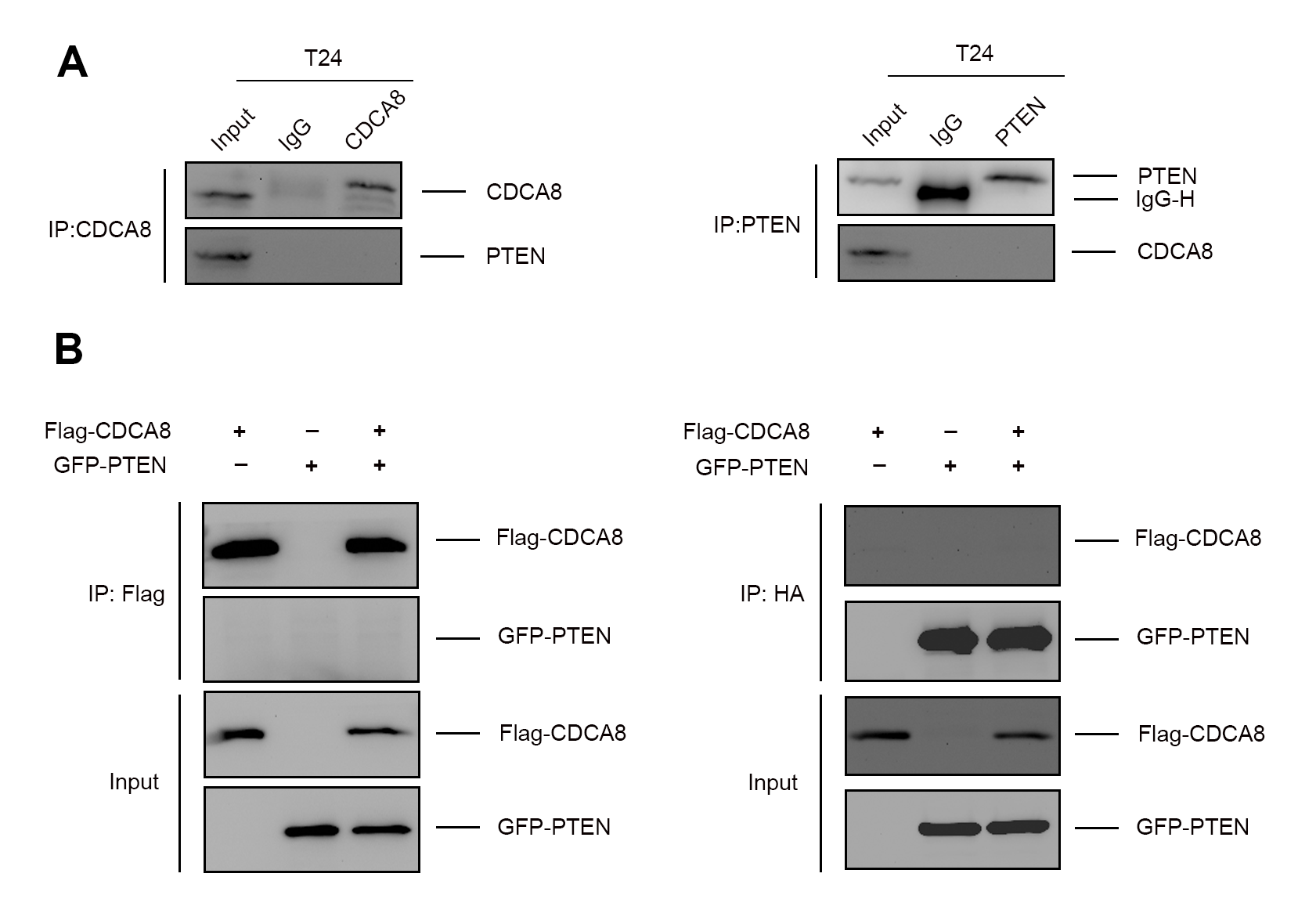
**

**Supplementary Fig. S7 There was no interaction** **between CDCA8 and PTEN.** (A) Co-IP assay displayed no endogenous interaction between CDCA8 and PTEN in T24 cells. (B) Co-IP assay showed no exogenous interaction between CDCA8 and PTEN in 293T cell.


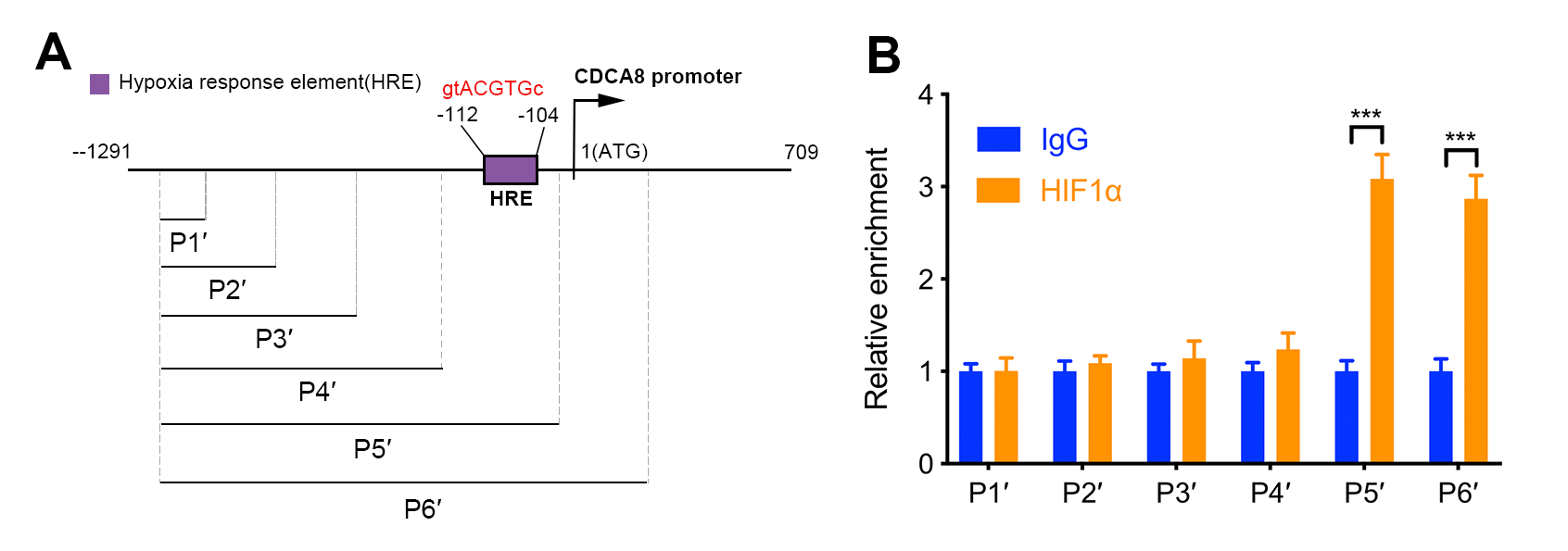


**Supplementary Fig. S8 The ChIP assay proved the binding site of HIF1α to CDCA8 promoter.** (A) Schematic drawing of the CDCA8 promoter with highlighted features. The promoter locus was marked as six parts, together with indicated primers for ChIP-qPCR assay. (B) ChIP analysis of BCa UMUC3 cells upon transfection of vector (control) or Flag-tagged HIF1α. Processed chromatin was next immunoprecipitated with anti-Flag-HIF1α antibody for purification and quantification by qPCR analysis.
