# Supplementary material for: CDCA8 promotes bladder cancer survival by stabilizing HIF1α expression under hypoxia
Source: Cell Death Dis. 2023 Oct 9;14(10):658. doi: 10.1038/s41419-023-06189-x (PMC10562466; doi:10.1038/s41419-023-06189-x)

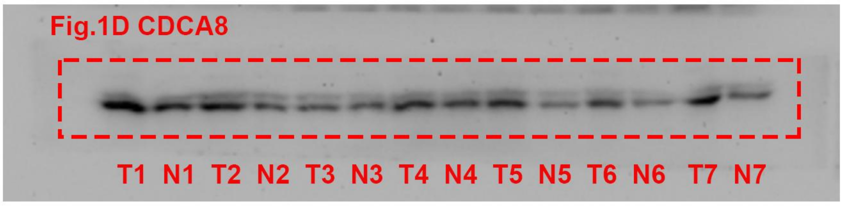

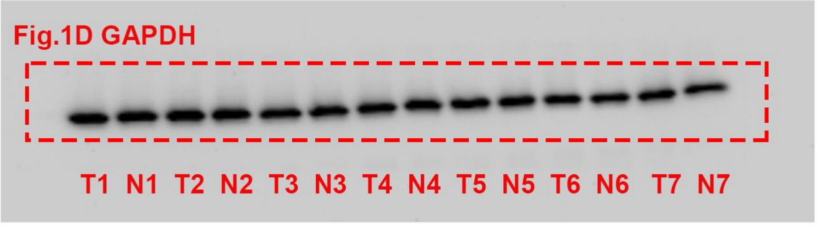

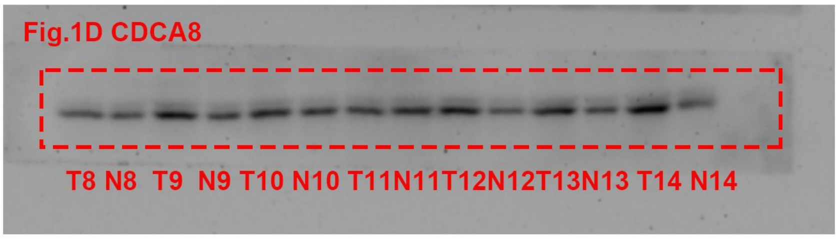

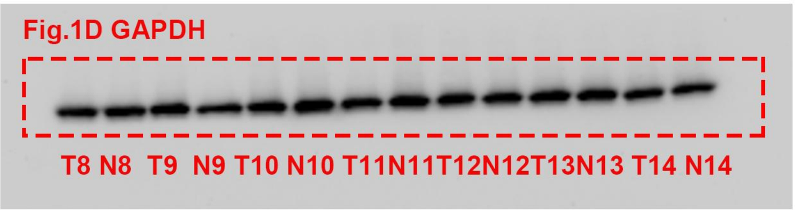

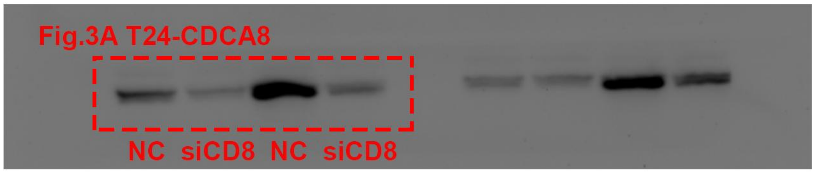

Fig.3A T24-HIF1α

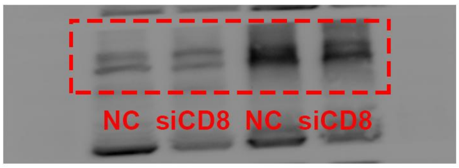

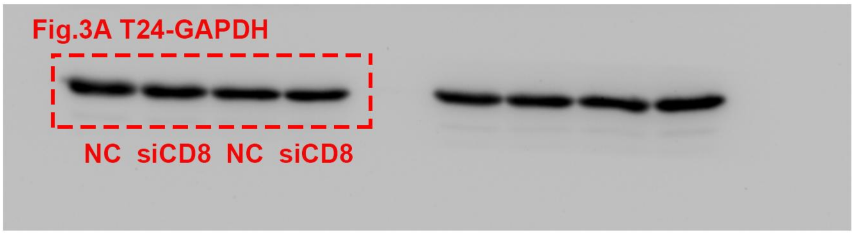

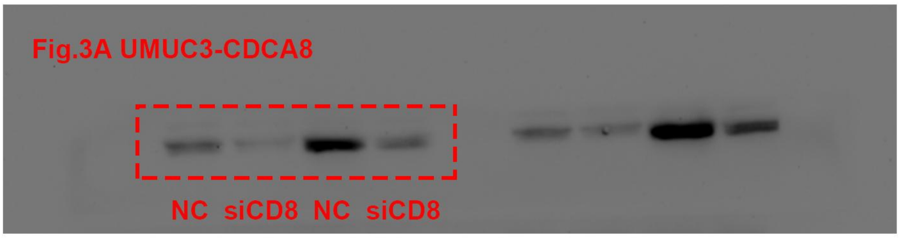

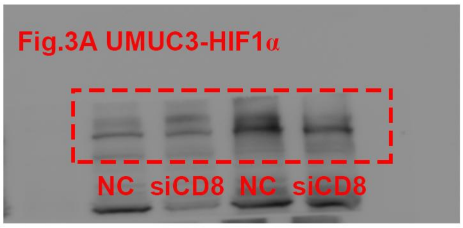

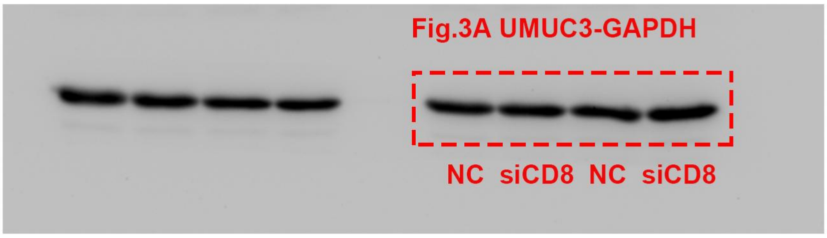

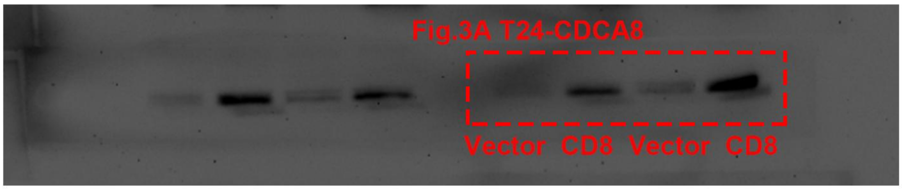
Fig.3A T24-CDCA8
Vector CD8 Vector CD8

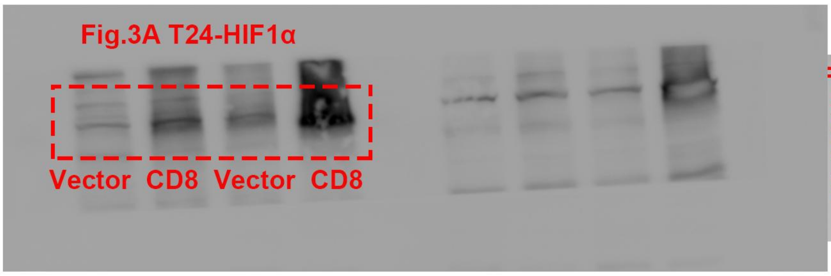
Fig.3A T24-HIF1α
Vector CD8 Vector CD8

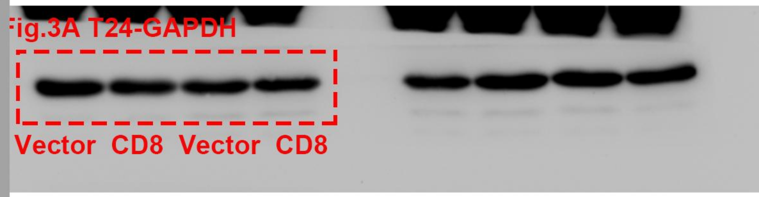
Fig.3A T24-GAPDH
Vector CD8 Vector CD8

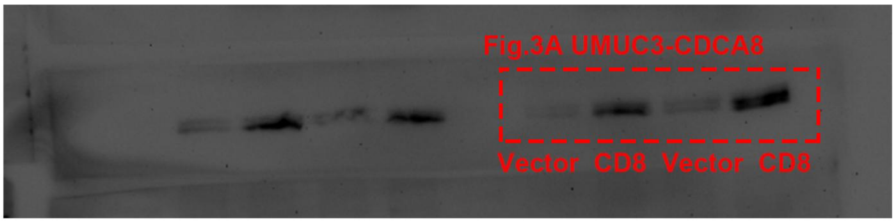
Fig.3A UMUC3-CDCA8
Vector CD8 Vector CD8

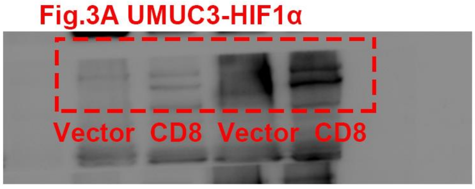
Fig.3A UMUC3-HIF1α
Vector CD8 Vector CD8

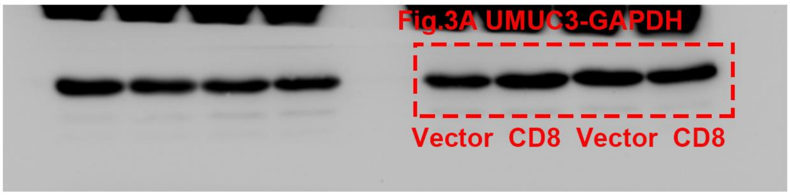
Fig.3A UMUC3-GAPDH
Vector CD8 Vector CD8

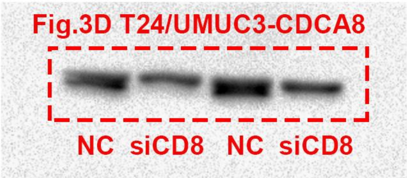
Fig.3D T24/UMUC3-CDCA8
NC siCD8 NC siCD8

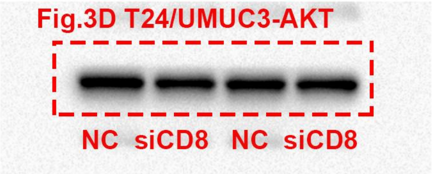
Fig.3D T24/UMUC3-AKT
NC siCD8 NC siCD8

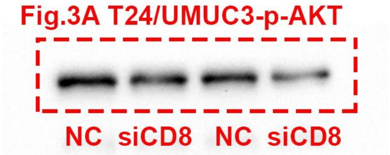
Fig.3A T24/UMUC3-p-AKT
NC siCD8 NC siCD8

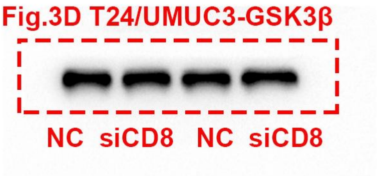
Fig.3D T24/UMUC3-GSK3β
NC siCD8 NC siCD8

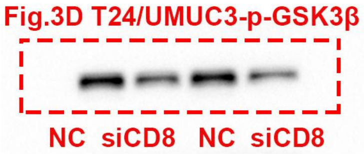
Fig.3D T24/UMUC3-p-GSK3β
NC siCD8 NC siCD8

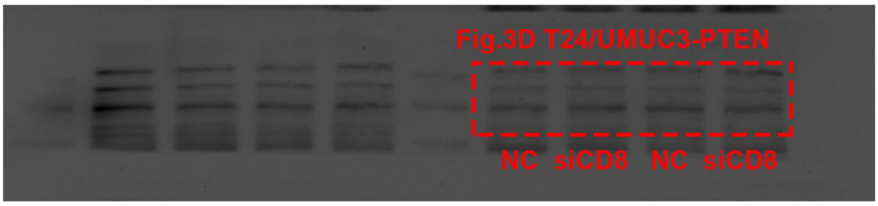
Fig.3D T24/UMUC3-PTEN
NC siCD8 NC siCD8

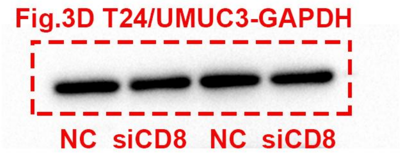
Fig.3D T24/UMUC3-GAPDH
NC siCD8 NC siCD8

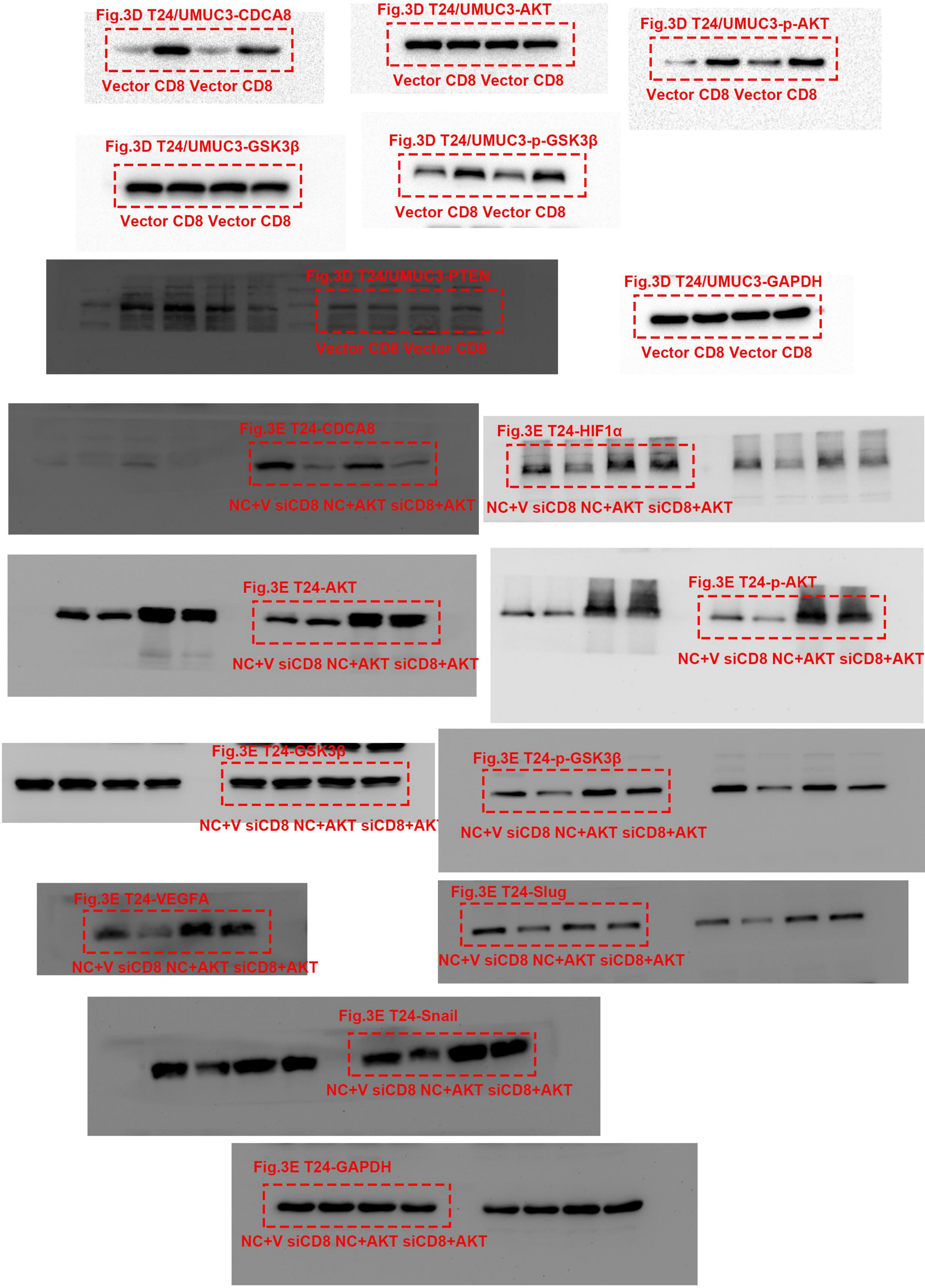
Fig.3D T24/UMUC3-CDCA8
Vector CD8 Vector CD8
Fig.3D T24/UMUC3-AKT
Vector CD8 Vector CD8
Fig.3D T24/UMUC3-p-AKT
Vector CD8 Vector CD8
Fig.3D T24/UMUC3-GSK3β
Vector CD8 Vector CD8
Fig.3D T24/UMUC3-p-GSK3β
Vector CD8 Vector CD8
Fig.3D T24/UMUC3-PTEN
Vector CD8 Vector CD8
Fig.3D T24/UMUC3-GAPDH
Vector CD8 Vector CD8
Fig.3E T24-CDCA8
NC+V siCD8 NC+AKT siCD8+AKT
Fig.3E T24-HIF1α
NC+V siCD8 NC+AKT siCD8+AKT
Fig.3E T24-AKT
NC+V siCD8 NC+AKT siCD8+AKT
Fig.3E T24-p-AKT
NC+V siCD8 NC+AKT siCD8+AKT
Fig.3E T24-GSK3β
NC+V siCD8 NC+AKT siCD8+AKT
Fig.3E T24-p-GSK3β
NC+V siCD8 NC+AKT siCD8+AKT
Fig.3E T24-VEGFA
NC+V siCD8 NC+AKT siCD8+AKT
Fig.3E T24-Slug
NC+V siCD8 NC+AKT siCD8+AKT
Fig.3E T24-Snail
NC+V siCD8 NC+AKT siCD8+AKT
Fig.3E T24-GAPDH
NC+V siCD8 NC+AKT siCD8+AKT

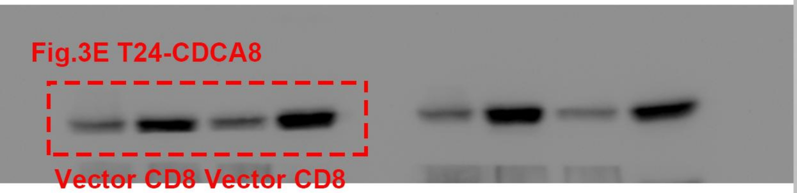
Fig.3E T24-CDCA8
Vector CD8 Vector CD8

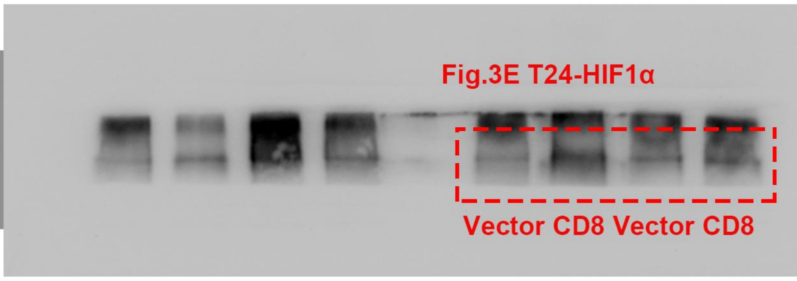
Fig.3E T24-HIF1α
Vector CD8 Vector CD8

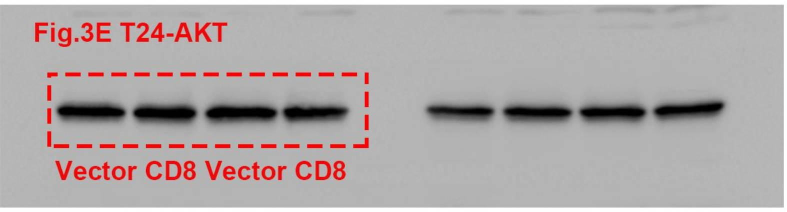
Fig.3E T24-AKT
Vector CD8 Vector CD8

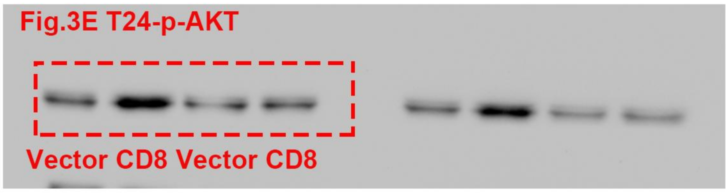
Fig.3E T24-p-AKT
Vector CD8 Vector CD8

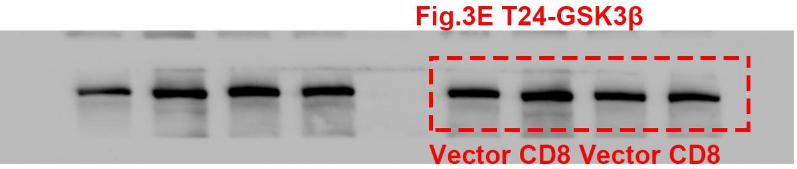
Fig.3E T24-GSK3β
Vector CD8 Vector CD8

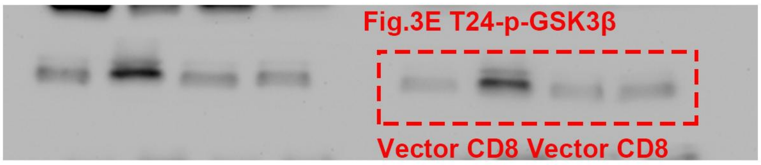
Fig.3E T24-p-GSK3β
Vector CD8 Vector CD8

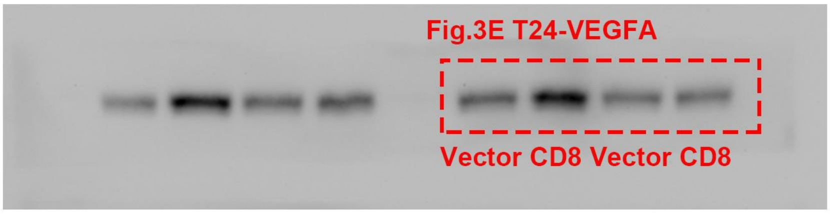
Fig.3E T24-VEGFA
Vector CD8 Vector CD8

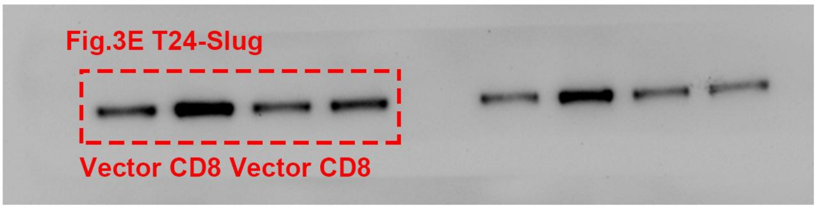
Fig.3E T24-Slug
Vector CD8 Vector CD8

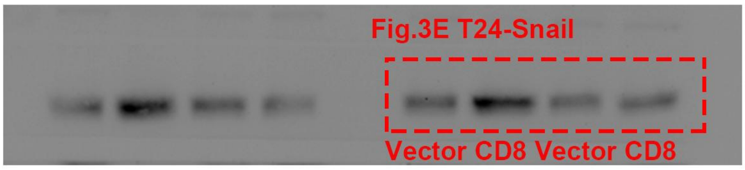
Fig.3E T24-Snail
Vector CD8 Vector CD8

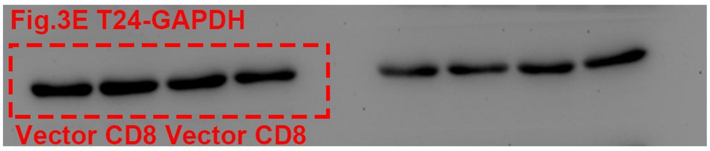
Fig.3E T24-GAPDH
Vector CD8 Vector CD8

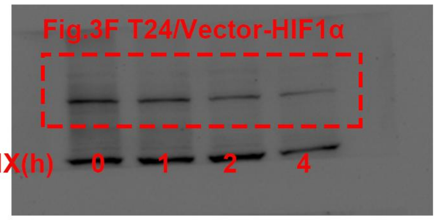
Fig.3F T24/Vector-HIF1α
CHX(h) 0 1 2 4

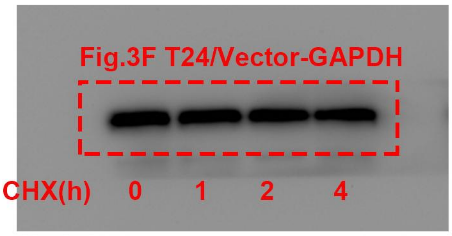
Fig.3F T24/Vector-GAPDH
CHX(h) 0 1 2 4

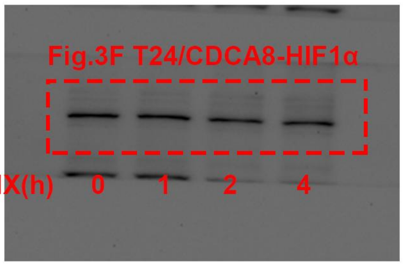
Fig.3F T24/CDCA8-HIF1α
CHX(h) 0 1 2 4

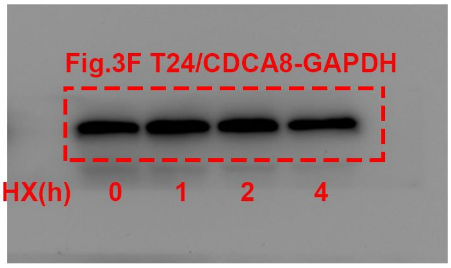
Fig.3F T24/CDCA8-GAPDH
CHX(h) 0 1 2 4

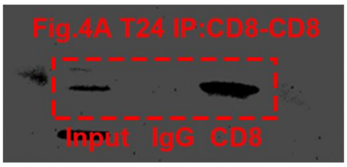
Fig.4A T24 IP:CD8-CD8
Input IgG CD8

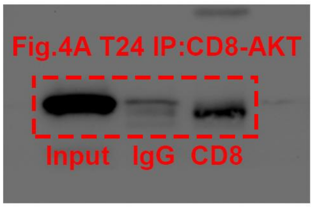
Fig.4A T24 IP:CD8-AKT
Input IgG CD8

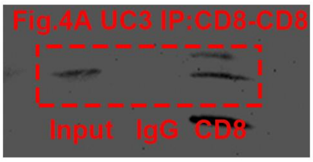
Fig.4A UC3 IP:CD8-CD8
Input IgG CD8

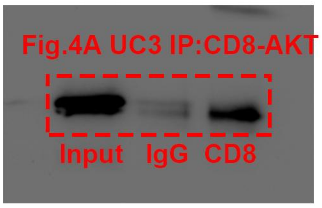
Fig.4A UC3 IP:CD8-AKT
Input IgG CD8

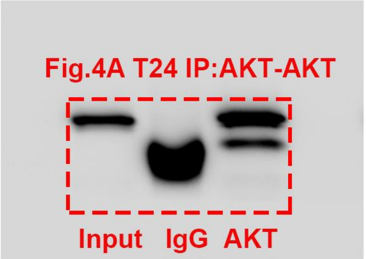
Fig.4A T24 IP:AKT-AKT
Input IgG AKT

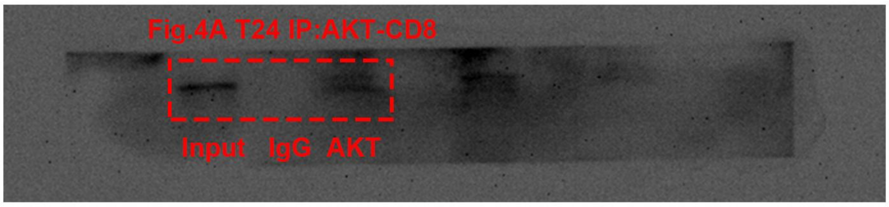
Fig.4A T24 IP:AKT-CD8
Input IgG AKT

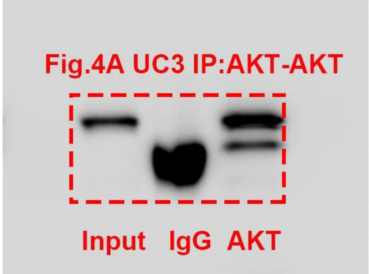
Fig.4A UC3 IP:AKT-AKT
Input IgG AKT

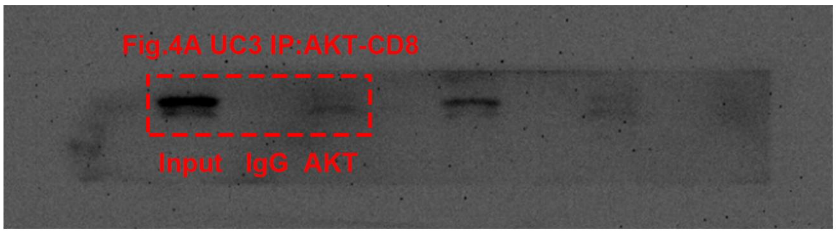
Fig.4A UC3 IP:AKT-CD8
Input IgG AKT

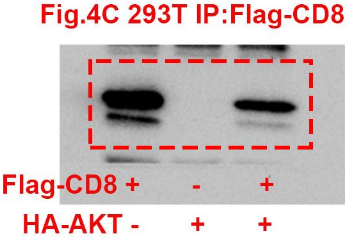
Fig.4C 293T IP:Flag-CD8
Flag-CD8 + - +
HA-AKT - + +

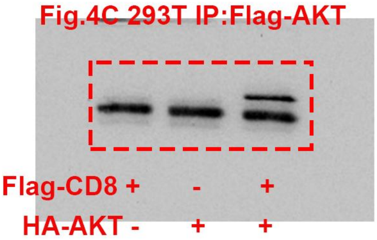
Fig.4C 293T IP:Flag-AKT
Flag-CD8 + - +
HA-AKT - + +

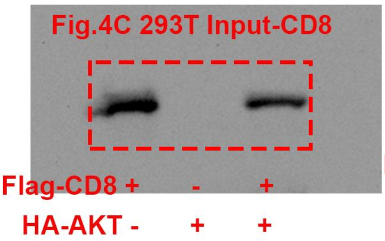
Fig.4C 293T Input-CD8
Flag-CD8 + - +
HA-AKT - + +

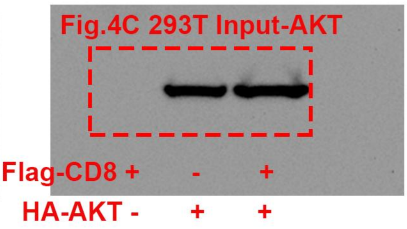
Fig.4C 293T Input-AKT
Flag-CD8 + - +
HA-AKT - + +

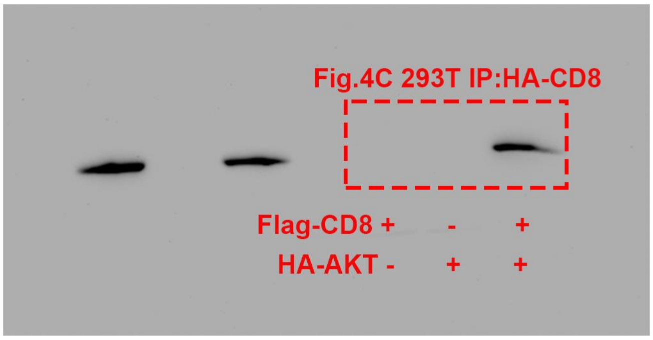
Fig.4C 293T IP:HA-CD8
Flag-CD8 + - +
HA-AKT - + +

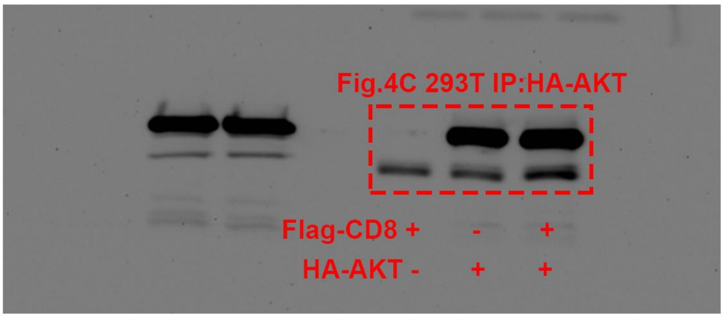
Fig.4C 293T IP:HA-AKT
Flag-CD8 + - +
HA-AKT - + +

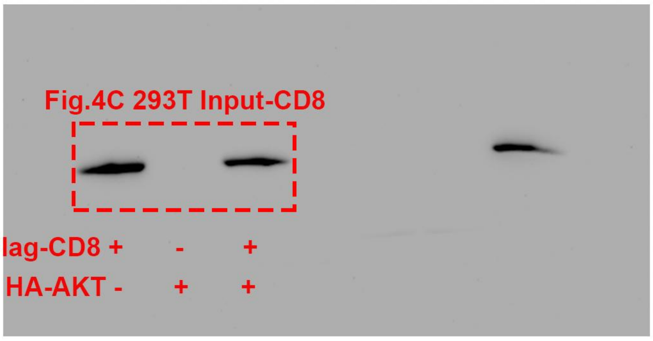
Fig.4C 293T Input-CD8
Flag-CD8 + - +
HA-AKT - + +

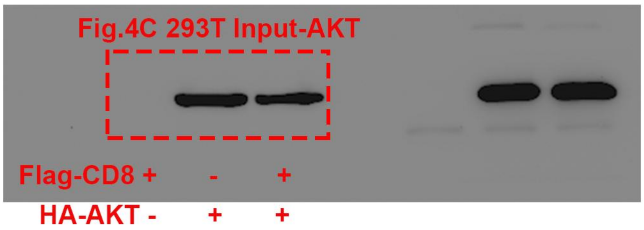
Fig.4C 293T Input-AKT
Flag-CD8 + - +
HA-AKT - + +

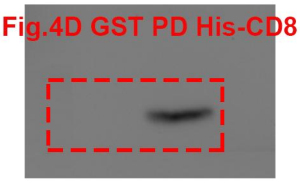
Fig.4D GST PD His-CD8
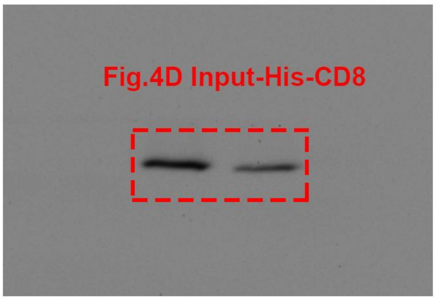
Fig.4D Input-His-CD8
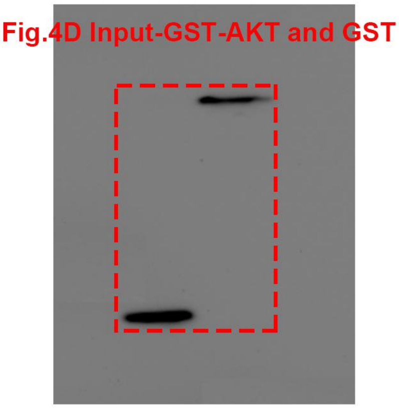
Fig.4D Input-GST-AKT and GST
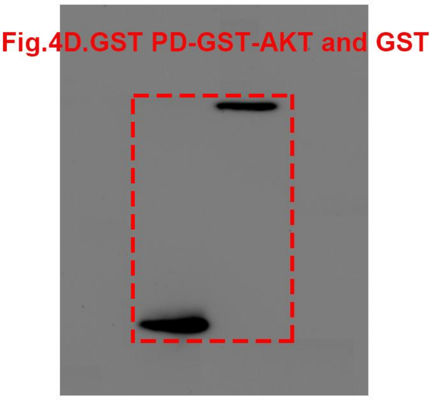
Fig.4D.GST PD-GST-AKT and GST
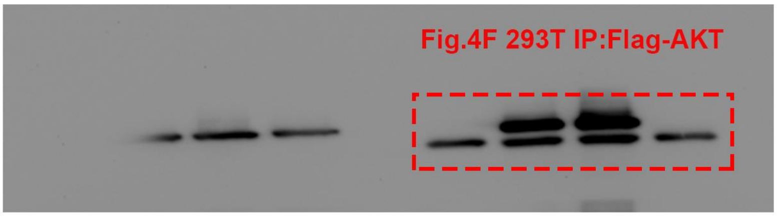
Fig.4F 293T IP:Flag-AKT
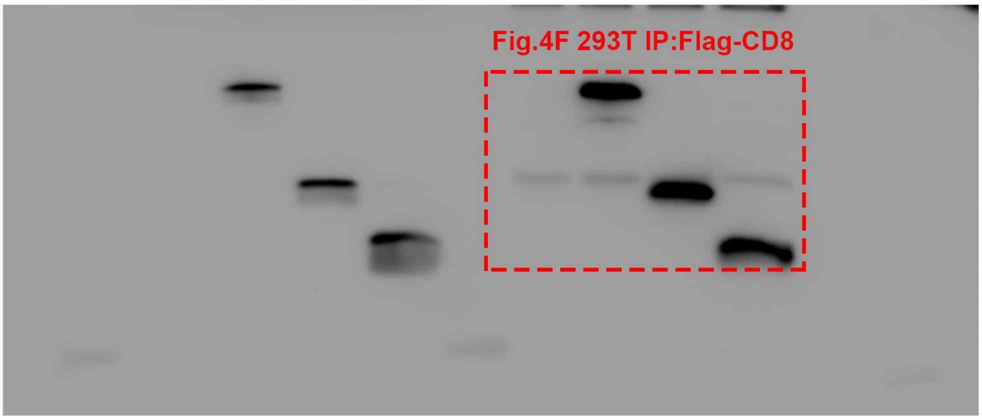
Fig.4F 293T IP:Flag-CD8
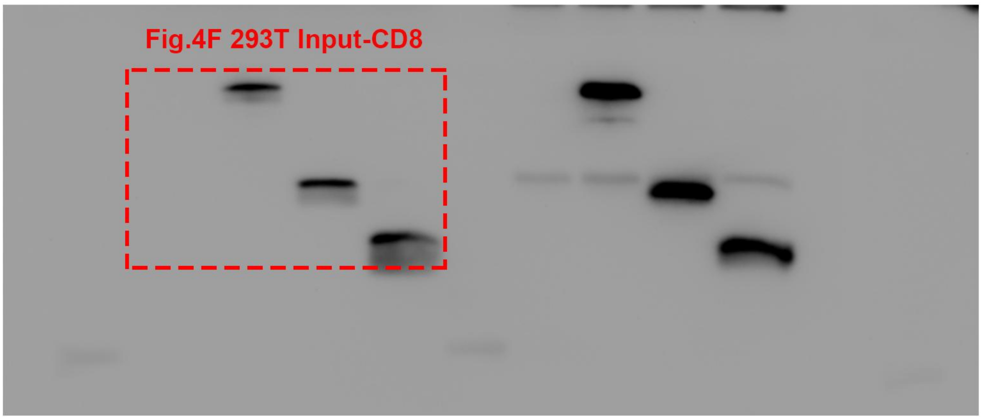
Fig.4F 293T Input-CD8
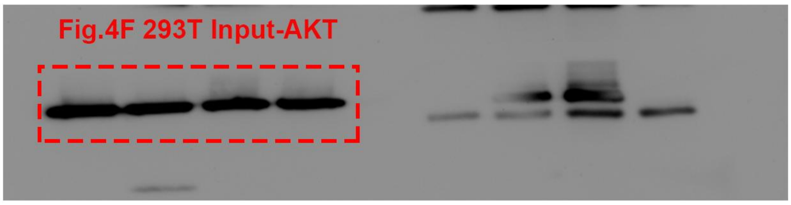
Fig.4F 293T Input-AKT

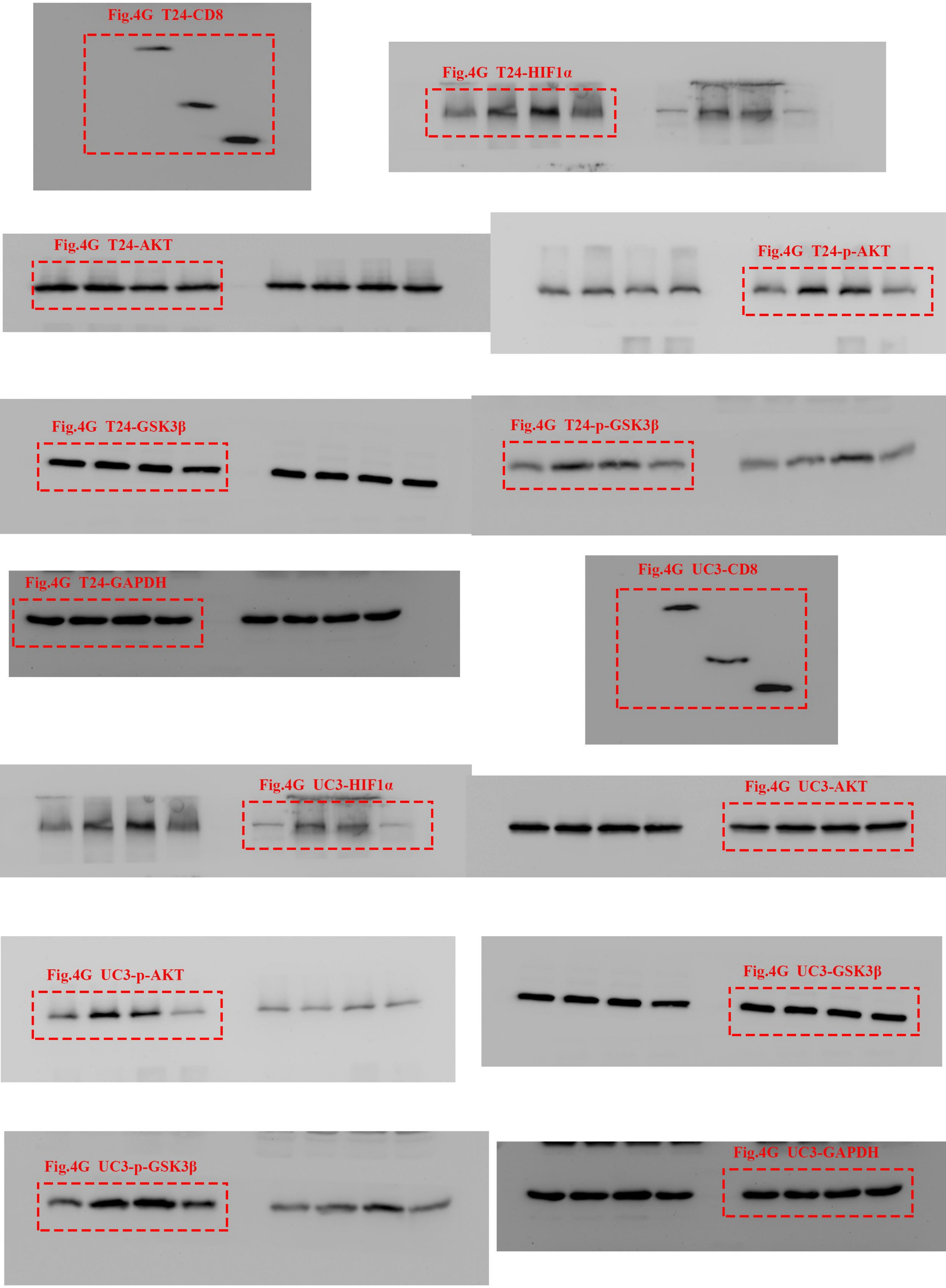
Fig.4G T24-CD8
Fig.4G T24-HIF1α
Fig.4G T24-AKT
Fig.4G T24-p-AKT
Fig.4G T24-GSK3β
Fig.4G T24-p-GSK3β
Fig.4G T24-GAPDH
Fig.4G UC3-CD8
Fig.4G UC3-HIF1α
Fig.4G UC3-AKT
Fig.4G UC3-p-AKT
Fig.4G UC3-GSK3β
Fig.4G UC3-p-GSK3β
Fig.4G UC3-GAPDH

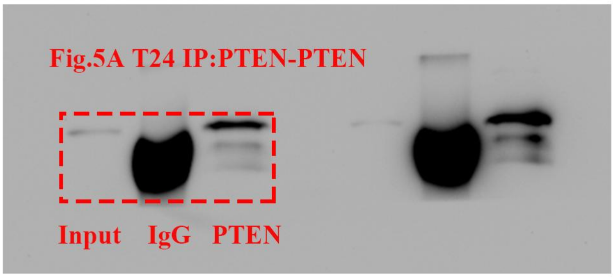
Fig.5A T24 IP:PTEN-PTEN
Input IgG PTEN

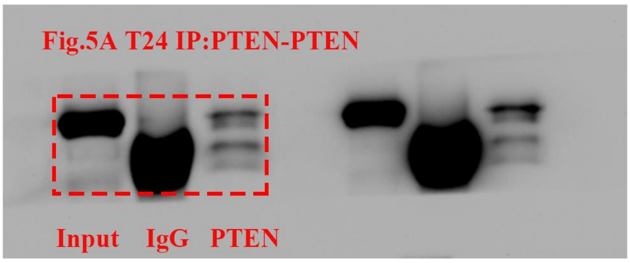
Fig.5A T24 IP:PTEN-PTEN
Input IgG PTEN

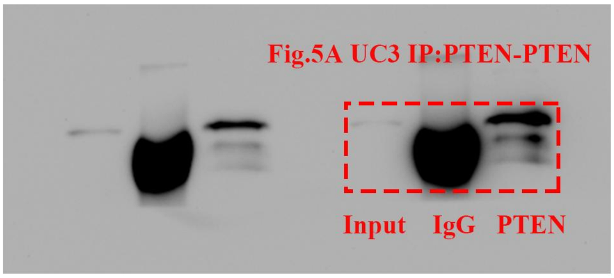
Fig.5A UC3 IP:PTEN-PTEN
Input IgG PTEN

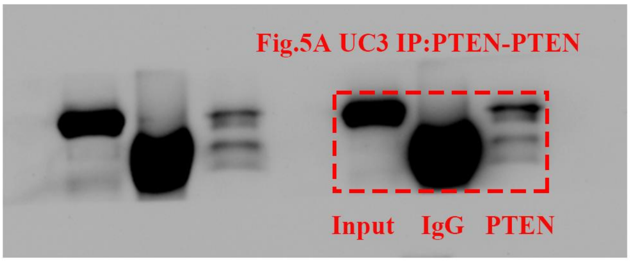
Fig.5A UC3 IP:PTEN-PTEN
Input IgG PTEN

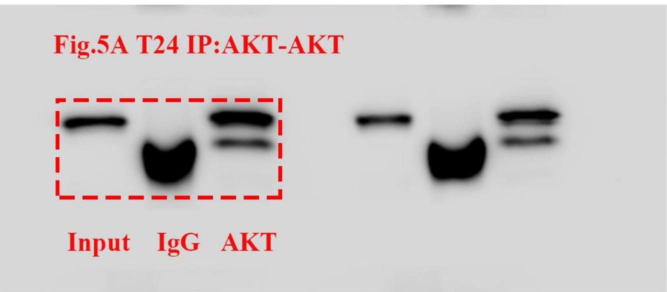
Fig.5A T24 IP:AKT-AKT
Input IgG AKT

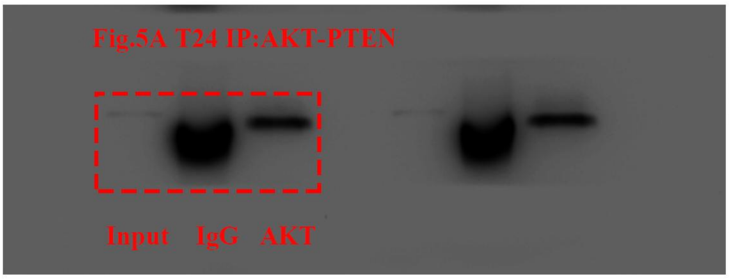
Fig.5A T24 IP:AKT-PTEN
Input IgG AKT

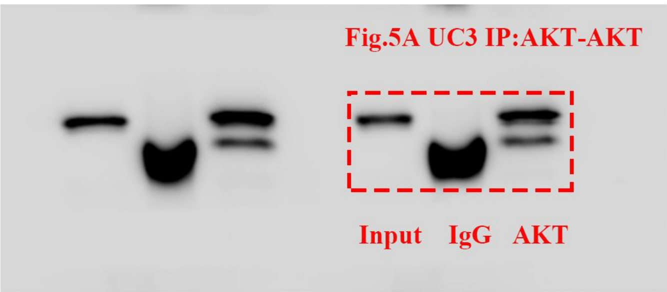
Fig.5A UC3 IP:AKT-AKT
Input IgG AKT

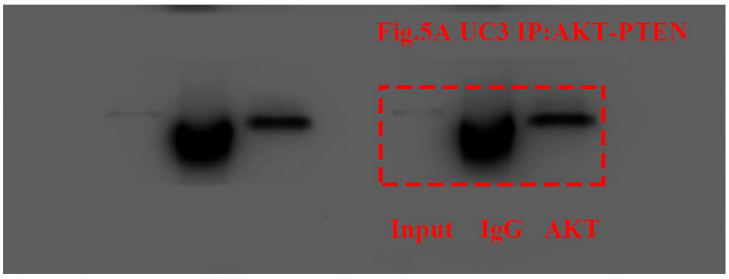
Fig.5A UC3 IP:AKT-PTEN
Input IgG AKT

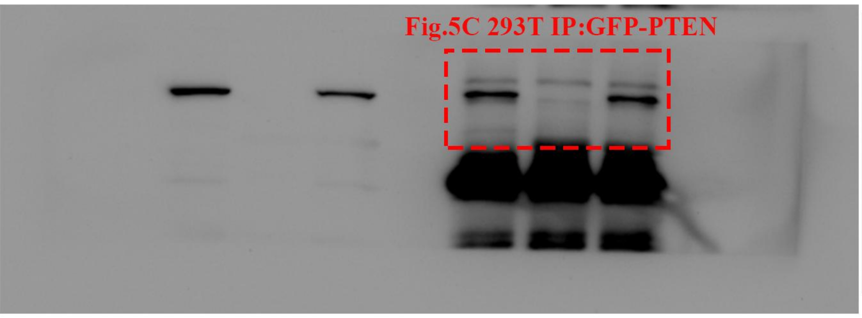
Fig.5C 293T IP:GFP-PTEN

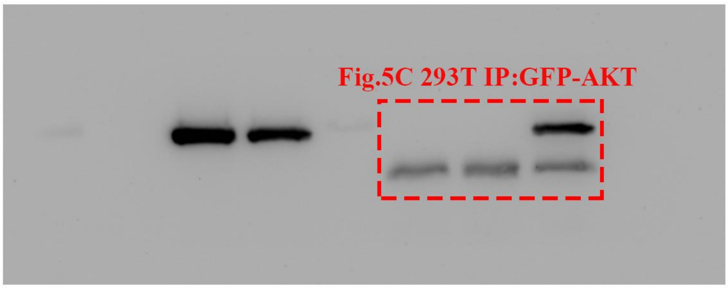
Fig.5C 293T IP:GFP-AKT

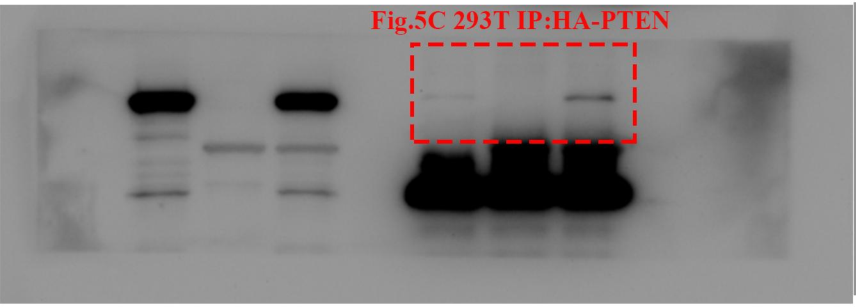
Fig.5C 293T IP:HA-PTEN

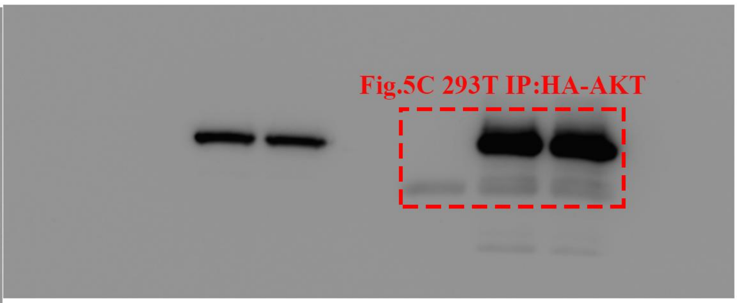
Fig.5C 293T IP:HA-AKT

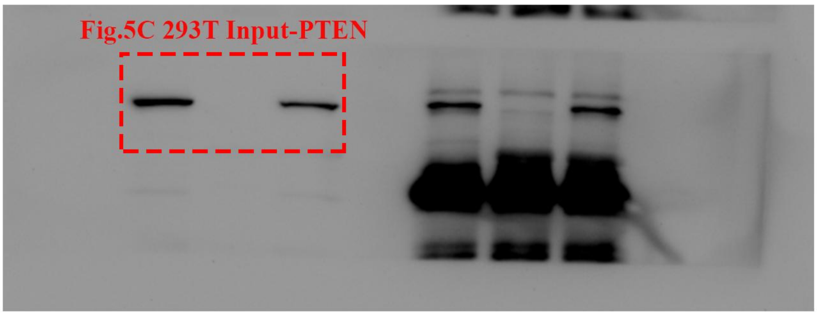
Fig.5C 293T Input-PTEN

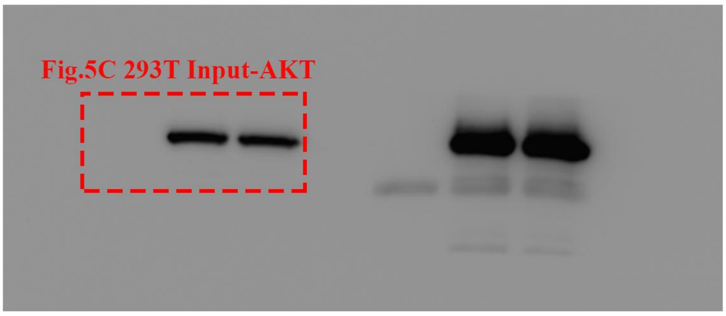
Fig.5C 293T Input-AKT

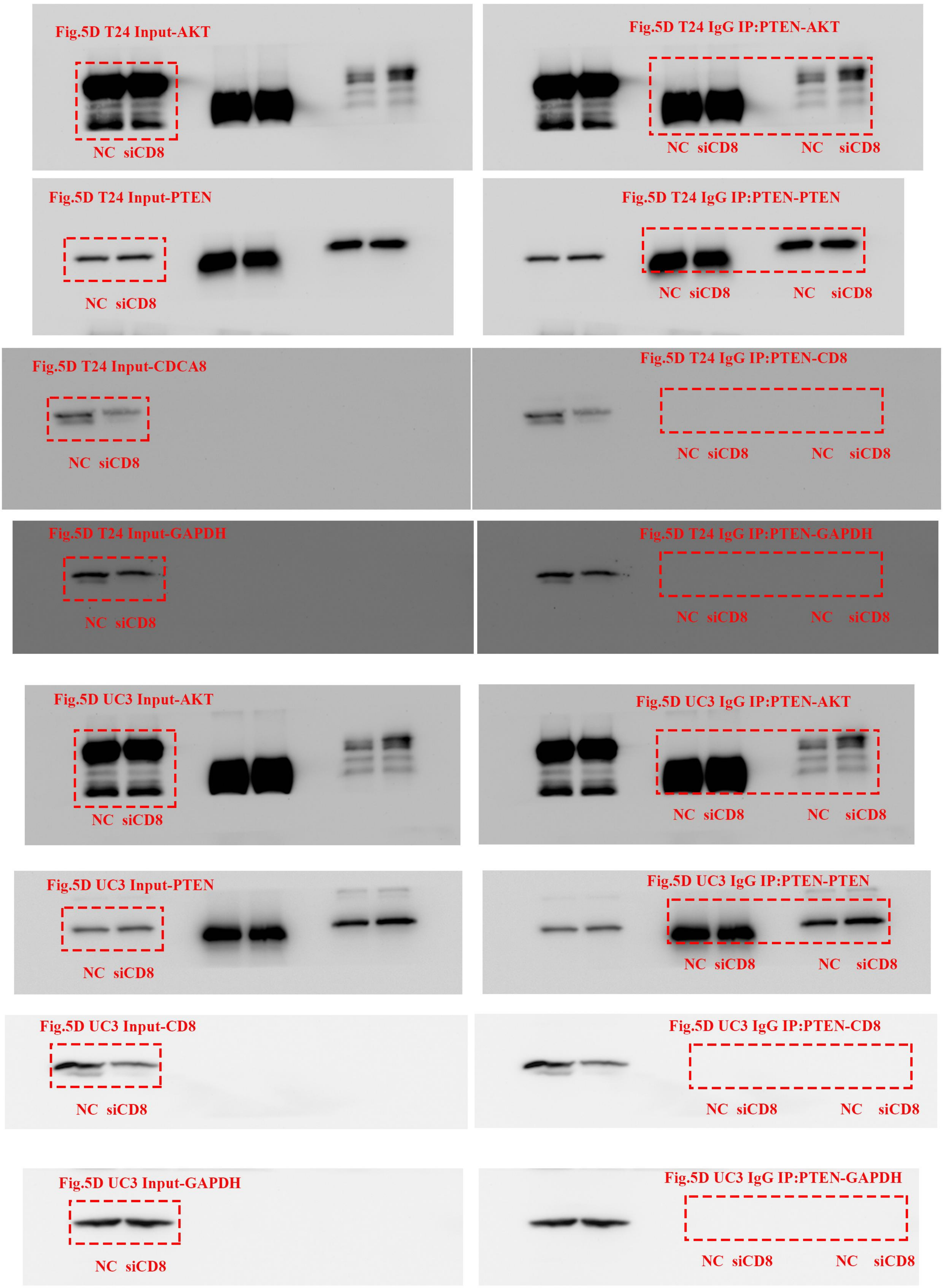
Fig.5D T24 Input-AKT
NC siCD8
Fig.5D T24 IgG IP:PTEN-AKT
NC siCD8 NC siCD8
Fig.5D T24 Input-PTEN
NC siCD8
Fig.5D T24 IgG IP:PTEN-PTEN
NC siCD8 NC siCD8
Fig.5D T24 Input-CDCA8
NC siCD8
Fig.5D T24 IgG IP:PTEN-CD8
NC siCD8 NC siCD8
Fig.5D T24 Input-GAPDH
NC siCD8
Fig.5D T24 IgG IP:PTEN-GAPDH
NC siCD8 NC siCD8
Fig.5D UC3 Input-AKT
NC siCD8
Fig.5D UC3 IgG IP:PTEN-AKT
NC siCD8 NC siCD8
Fig.5D UC3 Input-PTEN
NC siCD8
Fig.5D UC3 IgG IP:PTEN-PTEN
NC siCD8 NC siCD8
Fig.5D UC3 Input-CD8
NC siCD8
Fig.5D UC3 IgG IP:PTEN-CD8
NC siCD8 NC siCD8
Fig.5D UC3 Input-GAPDH
NC siCD8
Fig.5D UC3 IgG IP:PTEN-GAPDH
NC siCD8 NC siCD8

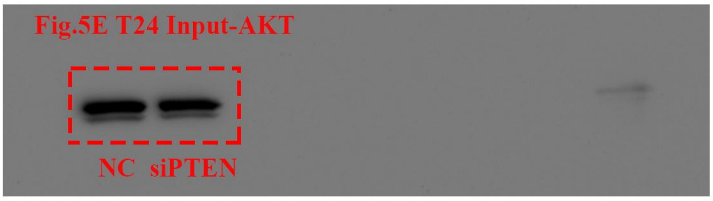
Fig.5E T24 Input-AKT
NC siPTEN

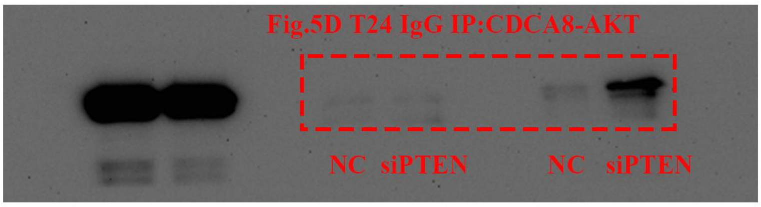
Fig.5D T24 IgG IP:CDCA8-AKT
NC siPTEN NC siPTEN

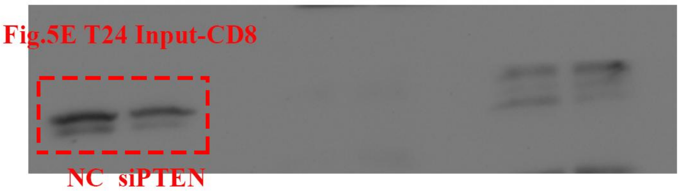
Fig.5E T24 Input-CD8
NC siPTEN

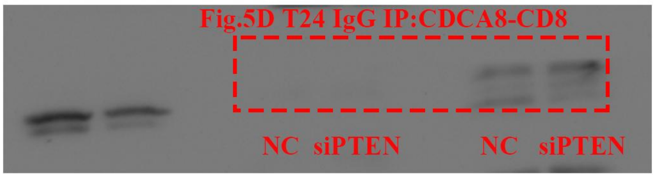
Fig.5D T24 IgG IP:CDCA8-CD8
NC siPTEN NC siPTEN

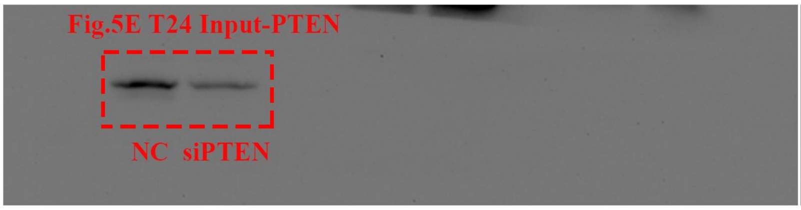
Fig.5E T24 Input-PTEN
NC siPTEN

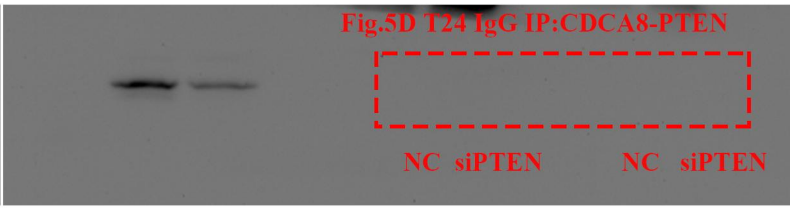
Fig.5D T24 IgG IP:CDCA8-PTEN
NC siPTEN NC siPTEN

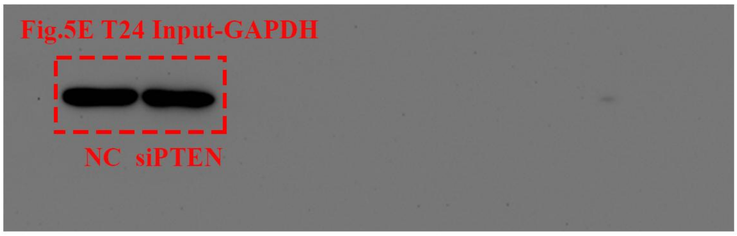
Fig.5E T24 Input-GAPDH
NC siPTEN

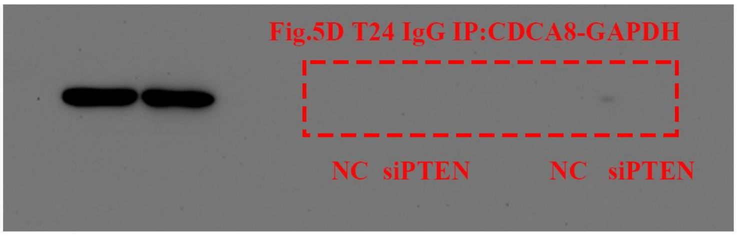
Fig.5D T24 IgG IP:CDCA8-GAPDH
NC siPTEN NC siPTEN

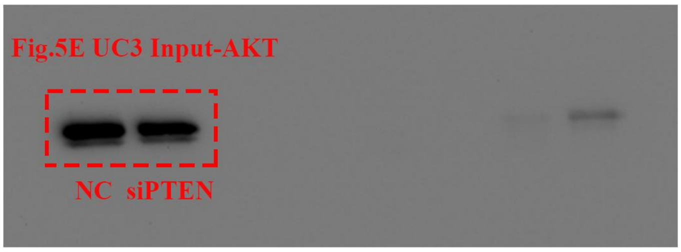
Fig.5E UC3 Input-AKT
NC siPTEN

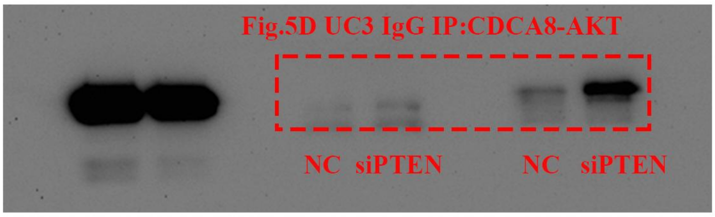
Fig.5D UC3 IgG IP:CDCA8-AKT
NC siPTEN NC siPTEN

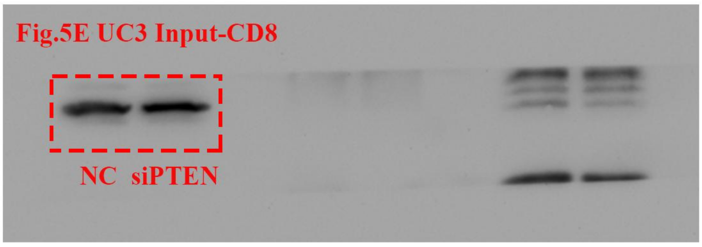
Fig.5E UC3 Input-CD8
NC siPTEN

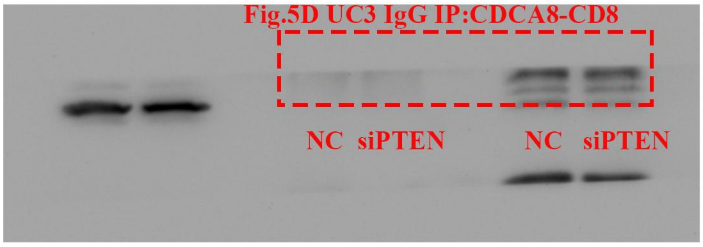
Fig.5D UC3 IgG IP:CDCA8-CD8
NC siPTEN NC siPTEN

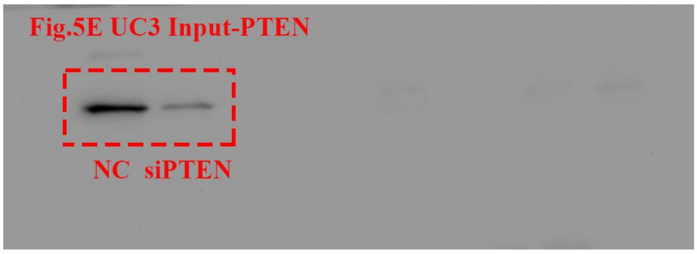
Fig.5E UC3 Input-PTEN
NC siPTEN

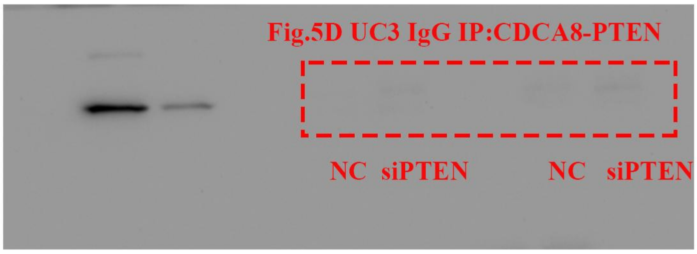
Fig.5D UC3 IgG IP:CDCA8-PTEN
NC siPTEN NC siPTEN

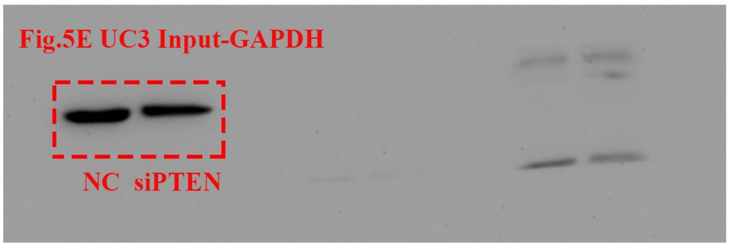
Fig.5E UC3 Input-GAPDH
NC siPTEN

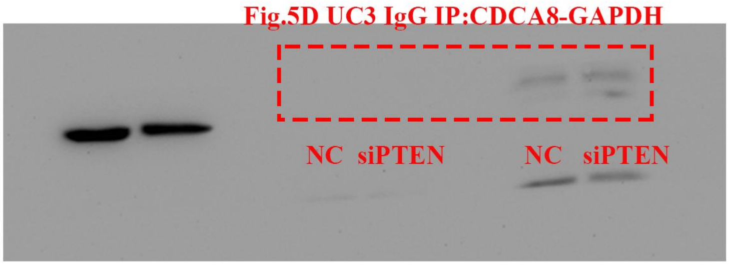
Fig.5D UC3 IgG IP:CDCA8-GAPDH
NC siPTEN NC siPTEN

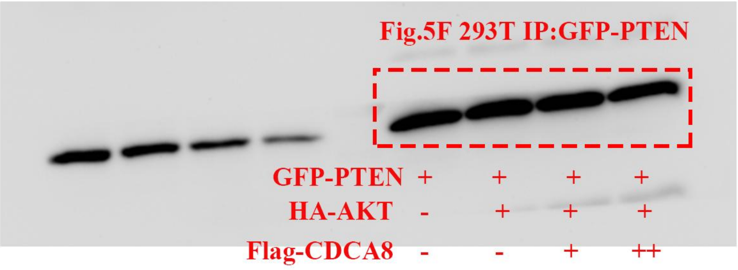
Fig.5F 293T IP:GFP-PTEN
GFP-PTEN + + + +
HA-AKT - + + +
Flag-CDCA8 - - + ++

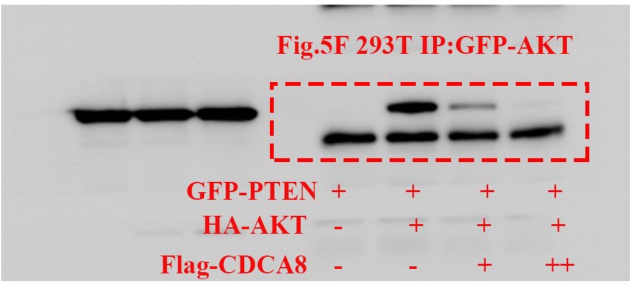
Fig.5F 293T IP:GFP-AKT
GFP-PTEN + + + +
HA-AKT - + + +
Flag-CDCA8 - - + ++

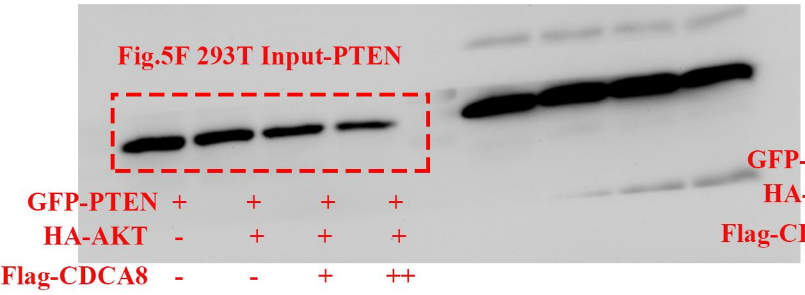
Fig.5F 293T Input-PTEN
GFP-PTEN + + + +
HA-AKT - + + +
Flag-CDCA8 - - + ++

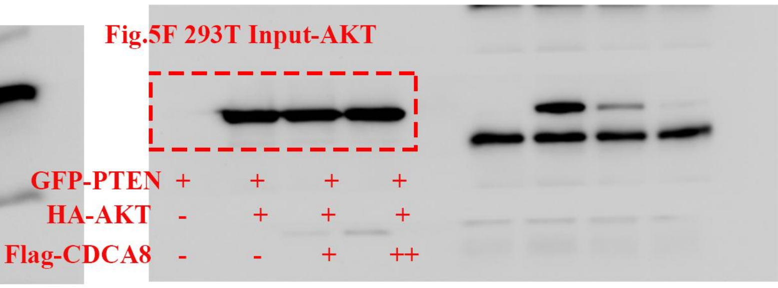
Fig.5F 293T Input-AKT
GFP-PTEN + + + +
HA-AKT - + + +
Flag-CDCA8 - - + ++

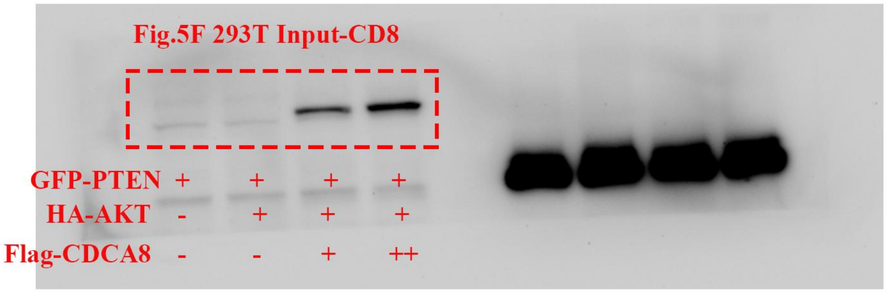
Fig.5F 293T Input-CD8
GFP-PTEN + + + +
HA-AKT - + + +
Flag-CDCA8 - - + ++

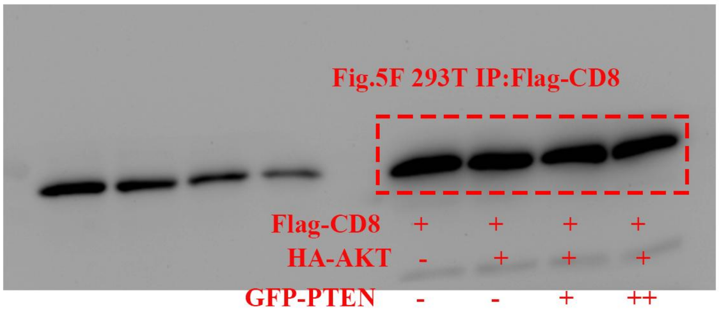
Fig.5F 293T IP:Flag-CD8
Flag-CD8 + + + +
HA-AKT - + + +
GFP-PTEN - - + ++

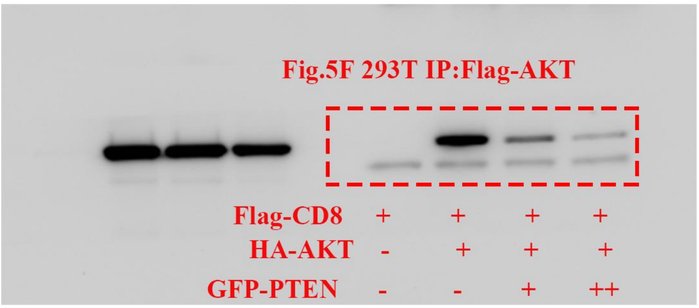
Fig.5F 293T IP:Flag-AKT
Flag-CD8 + + + +
HA-AKT - + + +
GFP-PTEN - - + ++

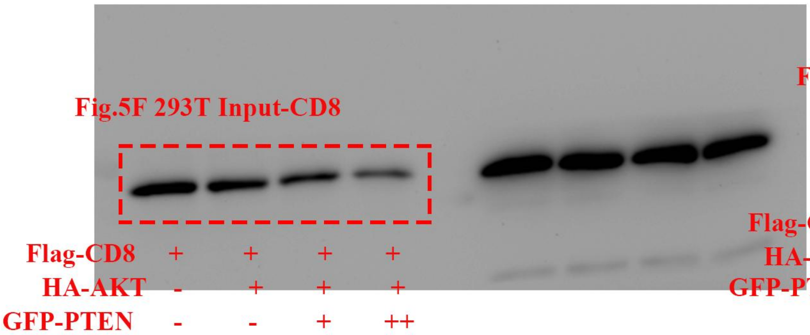
Fig.5F 293T Input-CD8
Flag-CD8 + + + +
HA-AKT - + + +
GFP-PTEN - - + ++

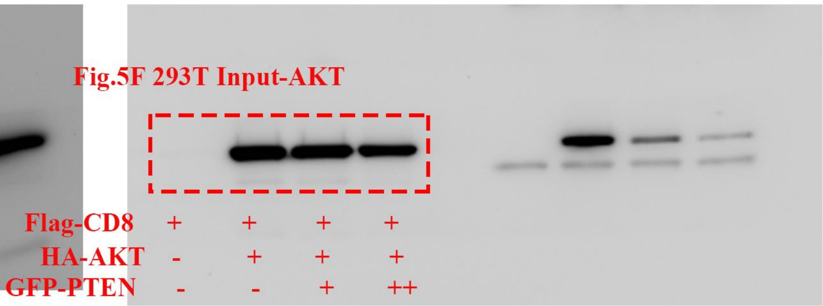
Fig.5F 293T Input-AKT
Flag-CD8 + + + +
HA-AKT - + + +
GFP-PTEN - - + ++

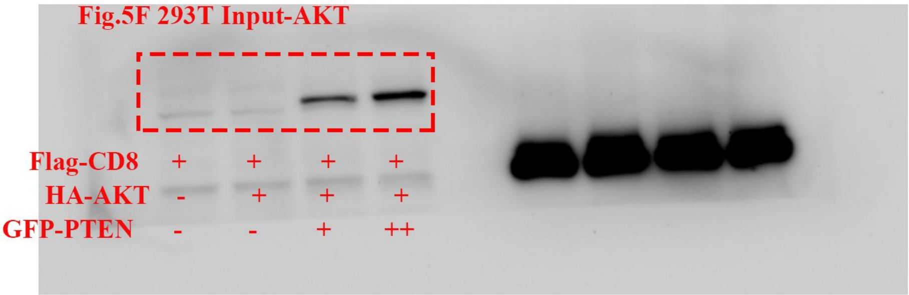
Fig.5F 293T Input-AKT
Flag-CD8 + + + +
HA-AKT - + + +
GFP-PTEN - - + ++

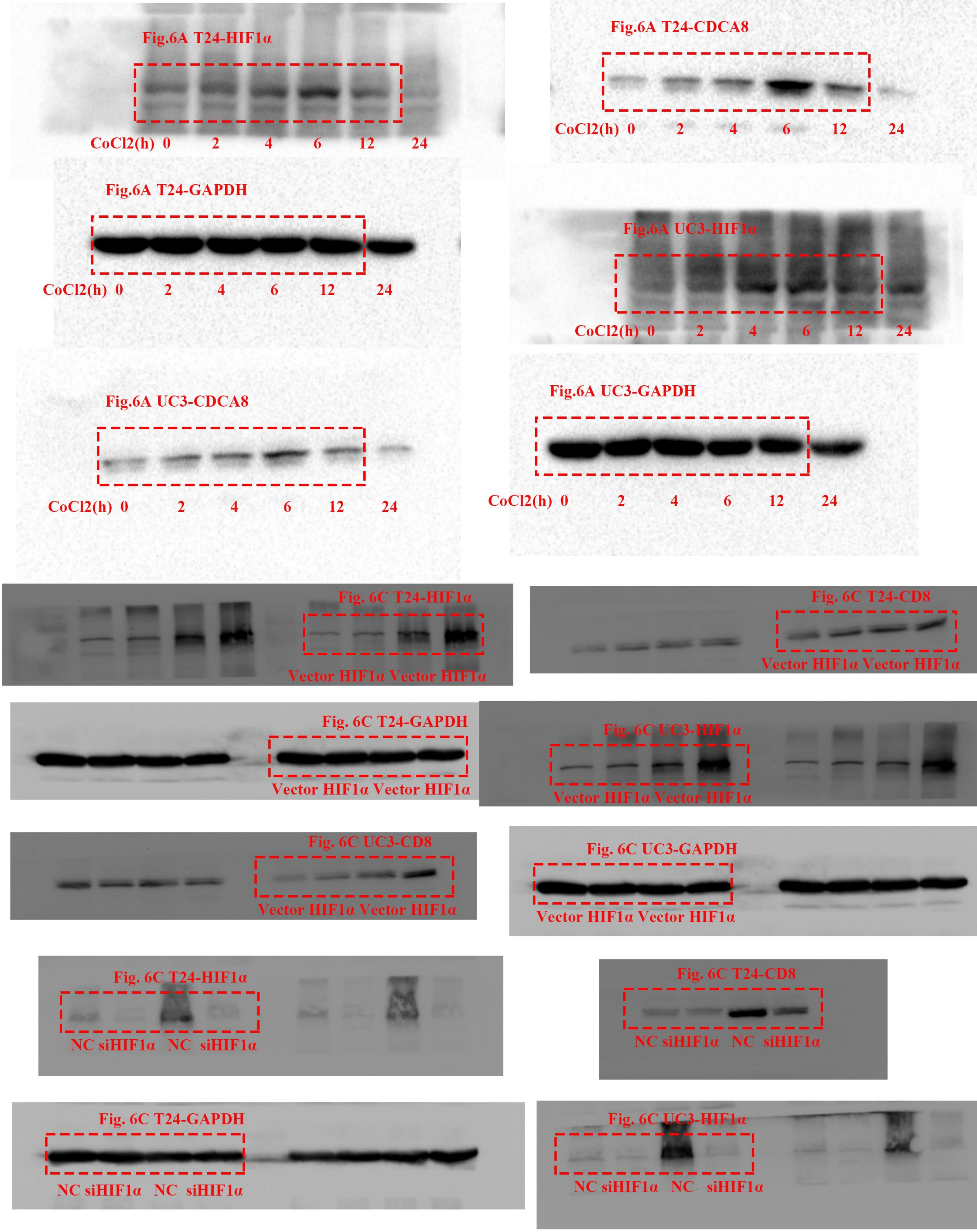

Fig.6A T24-HIF1α
CoCl2(h) 0 2 4 6 12 24
Fig.6A T24-CDCA8
CoCl2(h) 0 2 4 6 12 24
Fig.6A T24-GAPDH
CoCl2(h) 0 2 4 6 12 24
Fig.6A UC3-HIF1α
CoCl2(h) 0 2 4 6 12 24
Fig.6A UC3-CDCA8
CoCl2(h) 0 2 4 6 12 24
Fig.6A UC3-GAPDH
CoCl2(h) 0 2 4 6 12 24
Fig. 6C T24-HIF1α
Vector HIF1α Vector HIF1α
Fig. 6C T24-CD8
Vector HIF1α Vector HIF1α
Fig. 6C T24-GAPDH
Vector HIF1α Vector HIF1α
Fig. 6C UC3-HIF1α
Vector HIF1α Vector HIF1α
Fig. 6C UC3-CD8
Vector HIF1α Vector HIF1α
Fig. 6C UC3-GAPDH
Vector HIF1α Vector HIF1α
Fig. 6C T24-HIF1α
NC siHIF1α NC siHIF1α
Fig. 6C T24-CD8
NC siHIF1α NC siHIF1α
Fig. 6C T24-GAPDH
NC siHIF1α NC siHIF1α
Fig. 6C UC3-HIF1α
NC siHIF1α NC siHIF1α

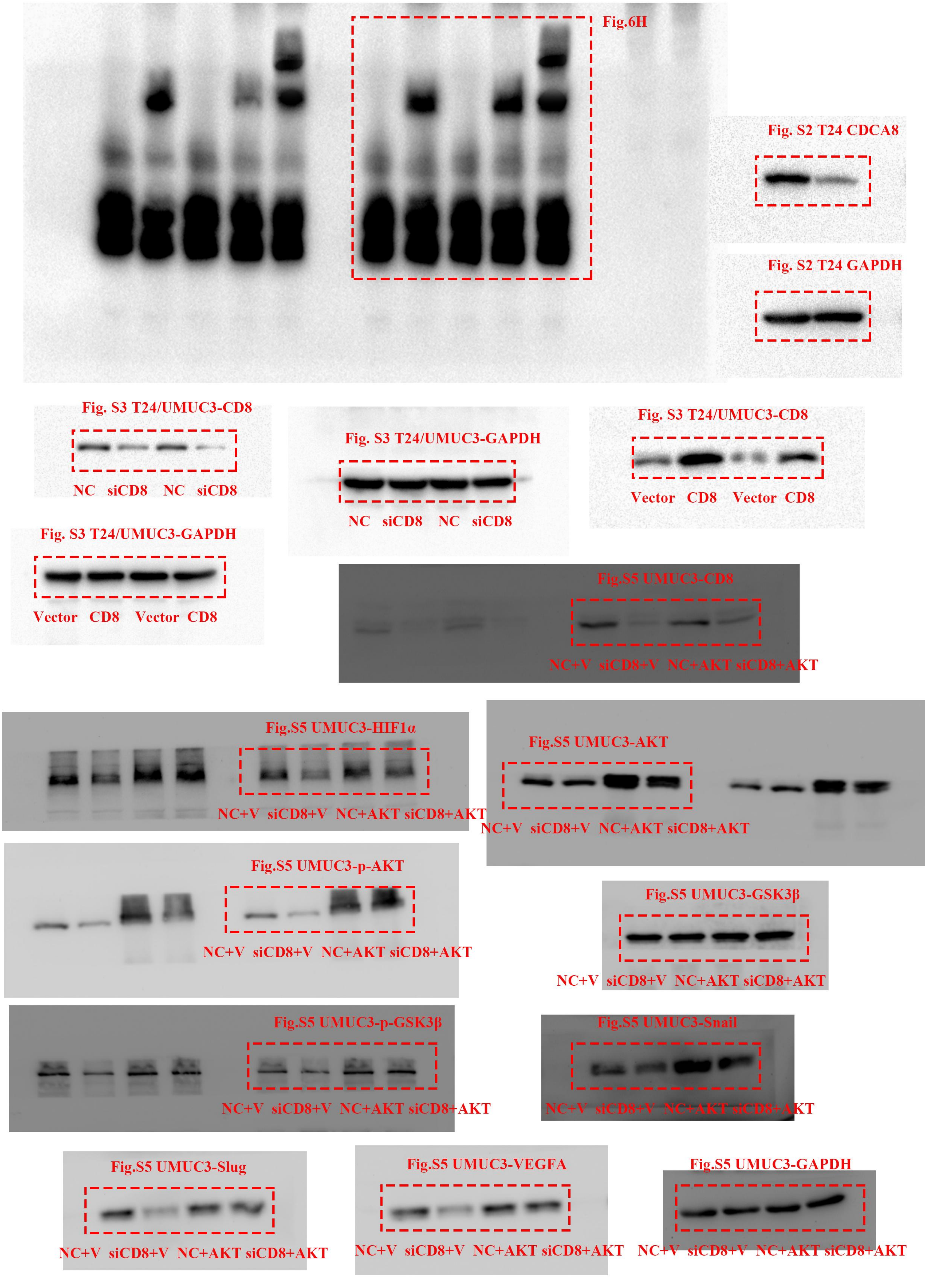

Fig.6H
Fig. S2 T24 CDCA8
Fig. S2 T24 GAPDH
Fig. S3 T24/UMUC3-CD8
NC siCD8 NC siCD8
Fig. S3 T24/UMUC3-GAPDH
NC siCD8 NC siCD8
Fig. S3 T24/UMUC3-CD8
Vector CD8 Vector CD8
Fig. S3 T24/UMUC3-GAPDH
Vector CD8 Vector CD8
Fig.S5 UMUC3-CD8
NC+V siCD8+V NC+AKT siCD8+AKT
Fig.S5 UMUC3-HIF1α
NC+V siCD8+V NC+AKT siCD8+AKT
Fig.S5 UMUC3-AKT
NC+V siCD8+V NC+AKT siCD8+AKT
Fig.S5 UMUC3-p-AKT
NC+V siCD8+V NC+AKT siCD8+AKT
Fig.S5 UMUC3-GSK3β
NC+V siCD8+V NC+AKT siCD8+AKT
Fig.S5 UMUC3-p-GSK3β
NC+V siCD8+V NC+AKT siCD8+AKT
Fig.S5 UMUC3-Snail
NC+V siCD8+V NC+AKT siCD8+AKT
Fig.S5 UMUC3-Slug
NC+V siCD8+V NC+AKT siCD8+AKT
Fig.S5 UMUC3-VEGFA
NC+V siCD8+V NC+AKT siCD8+AKT
Fig.S5 UMUC3-GAPDH
NC+V siCD8+V NC+AKT siCD8+AKT

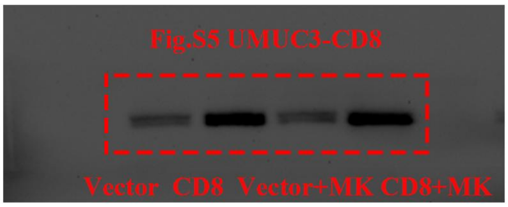
Fig.S5 UMUC3-CD8
Vector CD8 Vector+MK CD8+MK

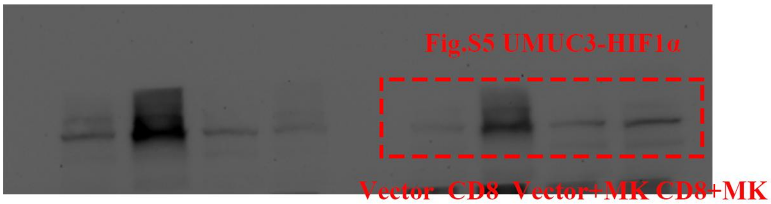
Fig.S5 UMUC3-HIF1α
Vector CD8 Vector+MK CD8+MK

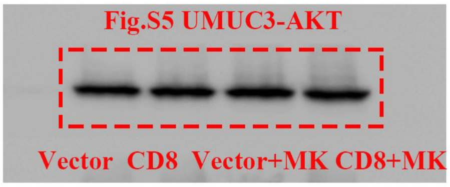
Fig.S5 UMUC3-AKT
Vector CD8 Vector+MK CD8+MK

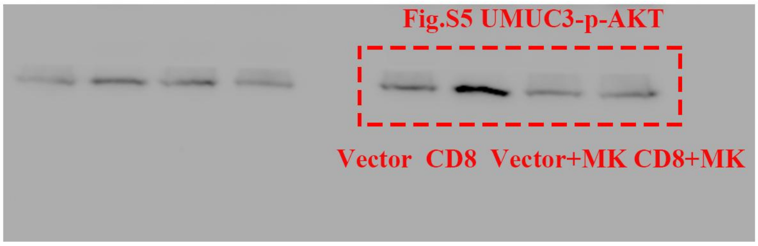
Fig.S5 UMUC3-p-AKT
Vector CD8 Vector+MK CD8+MK

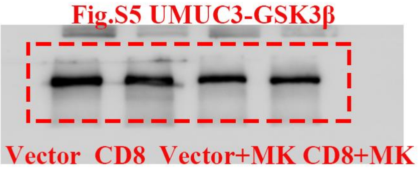
Fig.S5 UMUC3-GSK3β
Vector CD8 Vector+MK CD8+MK

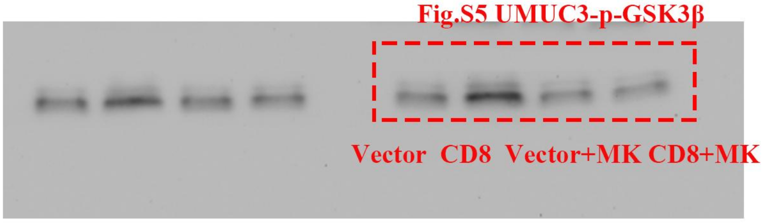
Fig.S5 UMUC3-p-GSK3β
Vector CD8 Vector+MK CD8+MK

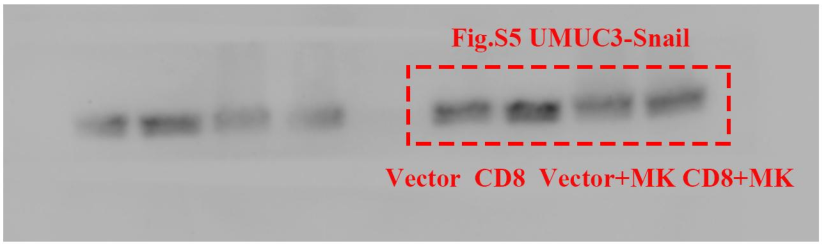
Fig.S5 UMUC3-Snail
Vector CD8 Vector+MK CD8+MK

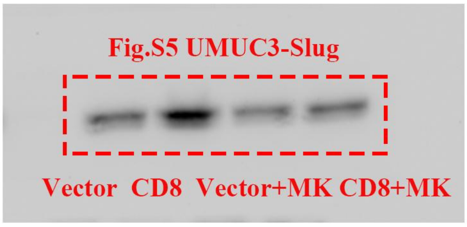
Fig.S5 UMUC3-Slug
Vector CD8 Vector+MK CD8+MK

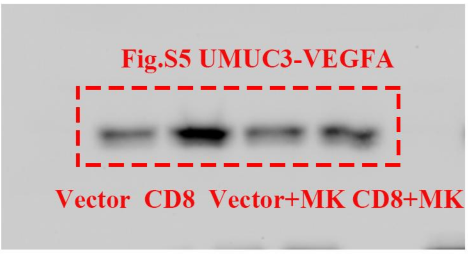
Fig.S5 UMUC3-VEGFA
Vector CD8 Vector+MK CD8+MK

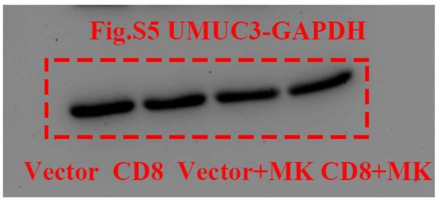
Fig.S5 UMUC3-GAPDH
Vector CD8 Vector+MK CD8+MK

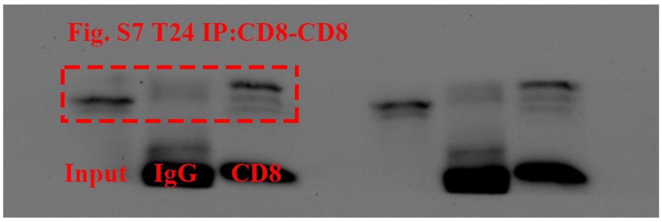
Fig. S7 T24 IP:CD8-CD8
Input IgG CD8

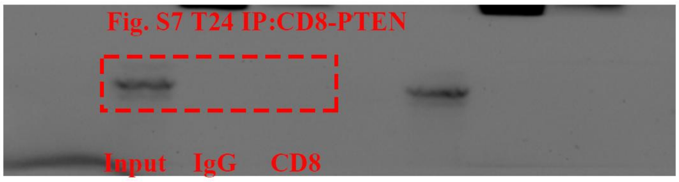
Fig. S7 T24 IP:CD8-PTEN
Input IgG CD8

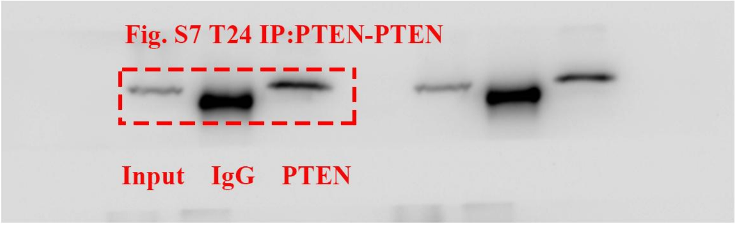
Fig. S7 T24 IP:PTEN-PTEN
Input IgG PTEN

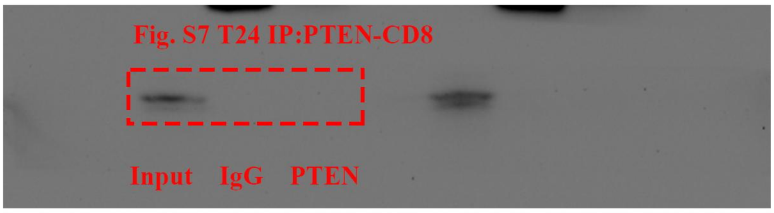
Fig. S7 T24 IP:PTEN-CD8
Input IgG PTEN

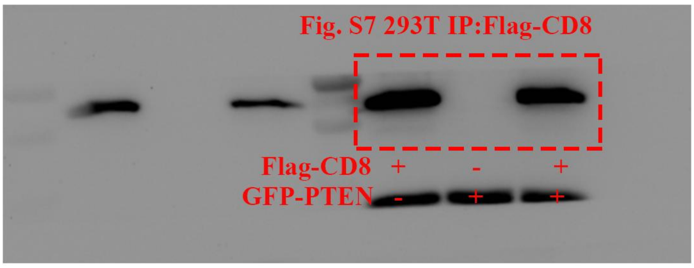

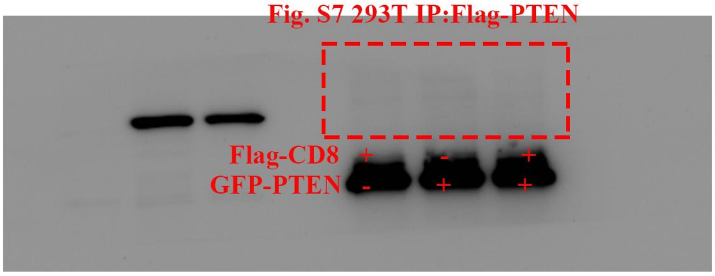

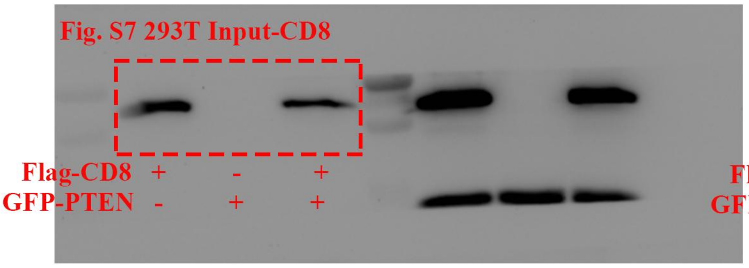

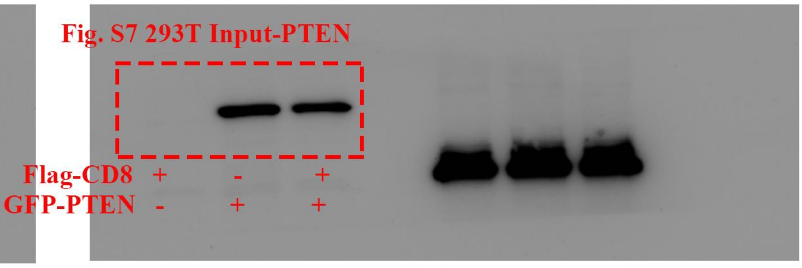

Fig. S7 293T IP:GFP-CD8

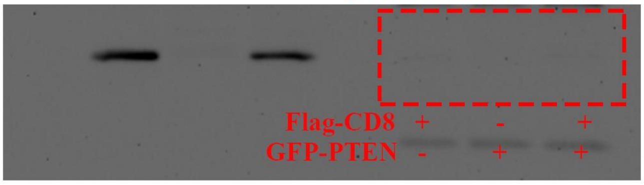

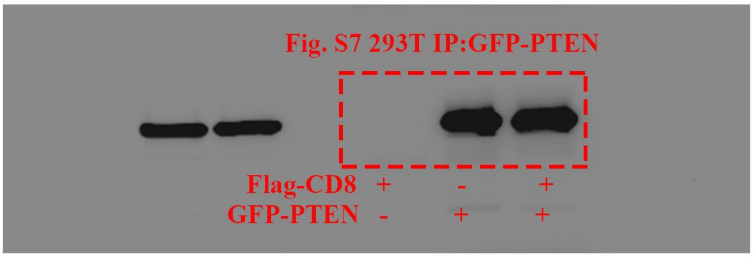

Fig. S7 293T Input-CD8

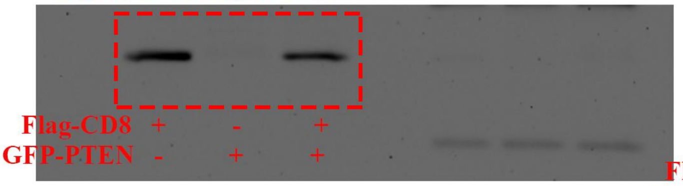

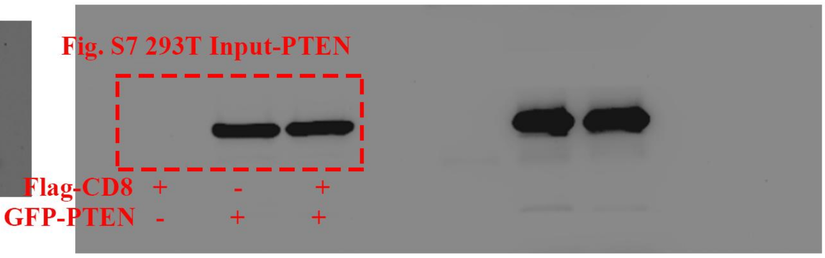

Supplement: Supplementary file 2 — Original Data File [file 41419_2023_6189_MOESM2_ESM.pdf]
